# Bolaamphiphile Analogues of 12-bis-THA Cl_2_ Are Potent Antimicrobial Therapeutics with Distinct Mechanisms of Action against Bacterial, Mycobacterial, and Fungal Pathogens

**DOI:** 10.1128/msphere.00508-22

**Published:** 2022-12-13

**Authors:** Simona Di Blasio, Maria Clarke, Charlotte K. Hind, Masanori Asai, Louis Laurence, Angelica Benvenuti, Mahnoor Hassan, Dorothy Semenya, DeDe Kwun-Wai Man, Victoria Horrocks, Giorgia Manzo, Sarah Van Der Lith, Carolyn Lam, Eugenio Gentile, Callum Annette, Janine Bosse, Yanwen Li, Barry Panaretou, Paul R. Langford, Brian D. Robertson, Jenny K. W. Lam, J. Mark Sutton, Michael McArthur, A. James Mason

**Affiliations:** a Institute of Pharmaceutical Science, School of Cancer & Pharmaceutical Sciences, King’s College London, London, United Kingdom; b Technology Development Group, UK Health Security Agency, Research and Evaluation, Salisbury, United Kingdom; c Section of Paediatric Infectious Disease, Department of Infectious Disease, Imperial College London, London, United Kingdom; d MRC Centre for Molecular Bacteriology and Infection, Department of Infectious Disease, Imperial College London, London, United Kingdom; e Norwich Medical School, University of East Anglia, Norwich, United Kingdom; f Department of Pharmacology & Pharmacy, Li Ka Shing Faculty of Medicine, The University of Hong Kong, Pokfulam, Hong Kong; g Department of Pharmaceutics, UCL School of Pharmacy, University College London, London, United Kingdom; JMI Laboratories

**Keywords:** dequalinium chloride, synergy, aminoglycosides, *Galleria mellonella*

## Abstract

12-Bis-THA Cl_2_ [12,12′-(dodecane-1,12-diyl)-bis-(9-amino-1,2,3,4-tetrahydroacridinium) chloride] is a cationic bolalipid adapted from dequalinium chloride (DQC), a bactericidal anti-infective indicated for bacterial vaginosis (BV). Here, we used a structure-activity-relationship study to show that the factors that determine effective killing of bacterial, fungal, and mycobacterial pathogens differ, to generate new analogues with a broader spectrum of activity, and to identify synergistic relationships, most notably with aminoglycosides against Acinetobacter baumannii and Pseudomonas aeruginosa, where the bactericidal killing rate was substantially increased. Like DQC, 12-bis-THA Cl_2_ and its analogues accumulate within bacteria and fungi. More hydrophobic analogues with larger headgroups show reduced potential for DNA binding but increased and broader spectrum antibacterial activity. In contrast, analogues with less bulky headgroups and stronger DNA binding affinity were more active against *Candida* spp. Shortening the interconnecting chain, from the most lipophilic twelve-carbon chain to six, improved the selectivity index against Mycobacterium tuberculosis *in vitro*, but only the longer chain analogue was therapeutic in a Galleria mellonella infection model, with the shorter chain analogue exacerbating the infection. *In vivo* therapy of Escherichia coli ATCC 25922 and epidemic methicillin-resistant Staphylococcus aureus 15 (EMRSA-15) infections in Galleria mellonella was also achieved with longer-chain analogues, as was therapy for an A. baumannii 17978 burn wound infection with a synergistic combination of bolaamphiphile and gentamicin. The present study shows how this class of bolalipids may be adapted further to enable a wider range of potential applications.

**IMPORTANCE** While we face an acute threat from antibiotic resistant bacteria and a lack of new classes of antibiotic, there are many effective antimicrobials which have limited application due to concerns regarding their toxicity and which could be more useful if such risks are reduced or eliminated. We modified a bolalipid antiseptic used in throat lozenges to see if it could be made more effective against some of the highest-priority bacteria and less toxic. We found that structural modifications that rendered the lipid more toxic against human cells made it less toxic in infection models and we could effectively treat caterpillars infected with either Mycobacterium tuberculosis, methicillin resistant Staphylococcus aureus, or Acinetobacter baumannii. The study provides a rationale for further adaptation toward diversifying the range of indications in which this class of antimicrobial may be used.

## INTRODUCTION

The stark prediction of the 2016 Review on Antimicrobial Resistance (1), that around 10 million deaths per year will be attributable to antimicrobial resistance (AMR) by the year 2050, has been reinforced by the recent finding that more than 1.2 million deaths in 2019 were a direct result of antibiotic-resistant bacterial infections (2). Actions recommended by the 2016 review to avert this include (i) identifying new antimicrobials that are effective against emerging drug-resistant bacteria and (ii) adopting more appropriate use of existing antimicrobials so their utility endures longer. Consistent with these two principles is the modification of existing antimicrobials to extend their range of applications and/or to mitigate the risk of resistance developing.

Bolaamphiphiles are amphipathic molecules that are composed of two hydrophilic groups at either end of an interconnecting hydrophobic skeleton chain (3). Notable examples from nature include lipids from archaebacteria, which form monolayered membranes, and, from the clinical setting, dequalinium chloride (DQC). DQC is composed of two quaternary quinolinium groups attached at either end of an *N*-decylene chain. It is available as throat lozenges (Dequadin) for treatment of sore throat and also thrush and glossitis, both notably associated with candidiasis, and is indicated for use as a topical treatment for bacterial vaginosis, formulated in tablets that are inserted at night (Fluomizin) (4). While DQC inhibits protein kinase C and is toxic toward mitochondria, it has many attractive features, including antibacterial, antifungal, antimalarial, and antitrypanosomal activity, while it is also an inhibitor of mycothiol ligase in Mycobacterium tuberculosis (5–7). Therefore, if the limitations that currently restrict the application of DQC can be better understood and overcome, there is scope for wider application of this class of antimicrobial.

12,12′-(Dodecane-1,12-diyl)-bis-(9-amino-1,2,3,4-tetrahydroacridinium) chloride (12-bis-THA Cl_2_, referred to here as CM2), was developed from a group of compounds, themselves developed by Weissig et al. from DQC (8), that varied in their self-assembly properties (9). Of these, 10-bis-THA, which includes a primary amine and bulky aliphatic residue in the headgroup and which was prepared as an iodide salt, formed the smallest vesicles with a narrow size distribution. Replacing iodide with chloride improves solubility and also self-assembly, and increasing the length of the interconnecting acyl chain from 10 to 12 carbons produced CM2, which has been used as an oligonucleotide nanovector (9). However, the CM2 headgroup, tacrine, is able to intercalate into DNA and is a topoisomerase inhibitor (10). Further development of CM2 toward use as an antimicrobial therefore hinges on evaluating the impact of its increased hydrophobicity and propensity to self-associate, relative to DQC, as well as investigating whether and how its intercalation and other undesirable properties may be mitigated.

Here, we describe a structure-activity relationship (SAR) approach that seeks to obtain a better understanding of the properties of cationic bolalipids that affect their antimicrobial activity and their potential to be used in a broader range of health care settings. The SAR study used CM2 as a starting point, and we investigated the ability of a series of analogues, with more or less bulky headgroups or with shorter acyl chains, to intercalate in DNA, their *in vitro* antibacterial and antifungal activities—including how synergy with existing antibiotics may alter their pharmacodynamic (PD) profile—and how the determinants of *in vitro* activity vary between microbial targets and also differ for successful therapy in Galleria mellonella infection models.

## RESULTS

### Analogue design and synthesis.

**FIG 1:**
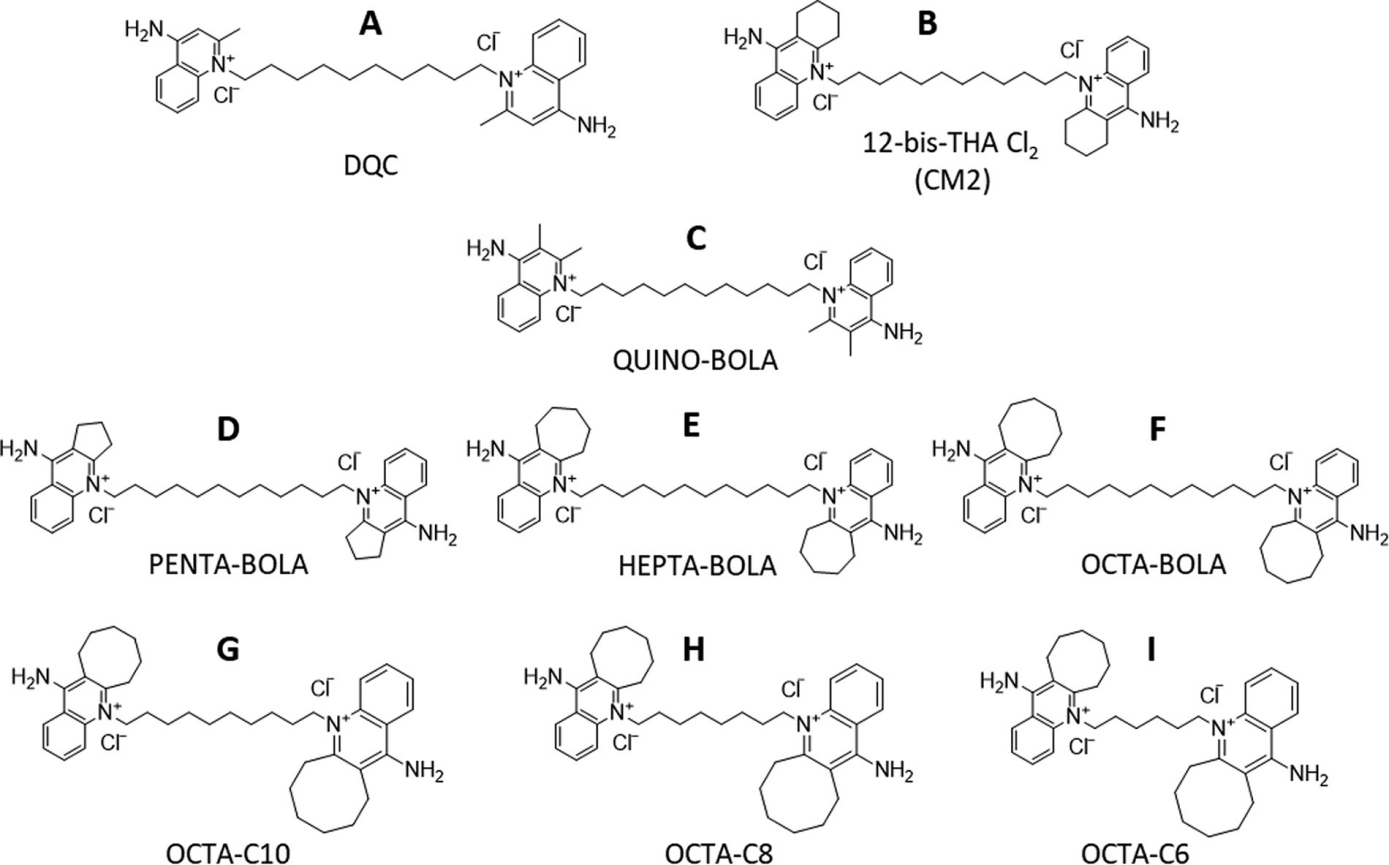
Design and naming of 12-bis-THA Cl_2_ analogues. Dequalinium chloride (A), CM2/12-bis-THA Cl_2_ (B), and the new bolalipids Quino-bola (C), Penta-bola (D), Hepta-bola (E), Octa-bola (F), Octa-C10 (G), Octa-C8 (H), and Octa-C6 (I), all prepared as chloride salts.

Because of the overarching aim to see if DNA intercalation and topoisomerase inhibition can be reduced while maintaining antimicrobial activity, seven new bolalipid analogues (Fig. 1C to I) were designed in which the bulkiness and planarity of the headgroup and/or the length of the acyl linker were varied. Each of these changes also impacts the overall hydrophobicity of the bolalipid, though the impacts of increasing the hydrophobicity of the headgroup or linker may differ. Starting with CM2 (Fig. 1B), the aliphatic region of the headgroup is either shrunk to from a six-carbon ring to either a five-carbon ring (Penta-bola) (Fig. 1D) or just two methyl groups (Quino-bola) (Fig. 1C) or enlarged to seven-carbon (Hepta-bola) (Fig. 1E) or eight-carbon (Octa-bola) (Fig. 1F) rings, which are bulkier and further disrupt the planarity of the headgroup. The rationale behind this strategy is based on the presence of free rotation around the N^+^-C bond: the bulky headgroups could be oriented in a way that could obstruct the headgroup and prevent it from inserting into DNA, whereas a less bulky one could facilitate the headgroup insertion between two successive DNA base pairs. Each of these analogues has the same twelve-carbon acyl linker.

Analogues with shorter acyl chains (Fig. 1G to I) compensate for the enlargement of the aliphatic region of the headgroup, which increases the overall hydrophobicity of the Octa-bola molecule relative to CM2. These analogues have either 10-carbon (Octa-C10) (Fig. 1G), eight-carbon (Octa-C8) (Fig. 1H), or six-carbon (Octa-C6) (Fig. 1I) acyl linkers.

Synthesis of the bolalipids is described in detail in the supplemental material. The four new headgroups were prepared with yields ranging from 45% (Penta) to 94% (Quino), and these compare favorably with tacrine, which in our hands was prepared at a yield of 61%. However, and as might be expected from previous studies of synthesis of gemini (*twin*) compounds based on either 4-aminopyridinium or 4-aminoquinaldinium headgroups (11), the tricyclic system may reduce the endocyclic nitrogen reactivity, and this is consistent with the relatively low yields (5 to 10%) obtained for the final bolalipids. Ion exchange was used to replace iodide and obtain the bolalipids as chloride salts.

### Increasing bolalipid headgroup bulkiness and shortening acyl linker reduces intercalation and topoisomerase I inhibition.

**FIG 2:**
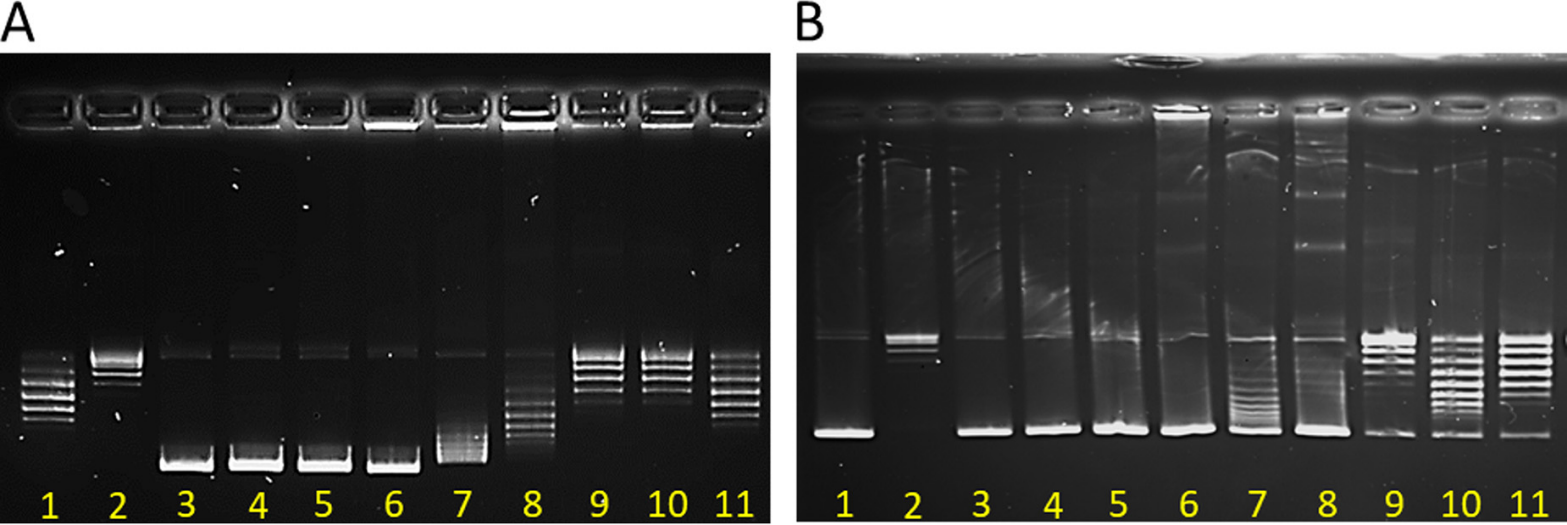
DNA intercalation is prevented by increasing bulkiness of the bolalipid headgroup. Unwinding assay gels are shown for relaxed (A) and supercoiled (B) pBR322 plasmid. In both gels, lane 1 contains plasmid only and lanes 2 to 11 contain plasmid plus topoisomerase I either alone (lane 2) or with DQC (lane 3), Quino-bola (lane 4), Penta-bola (lane 5), CM2 (lane 6), Hepta-bola (lane 7), Octa-bola (lane 8), Octa-C10 (lane 9), Octa-C8 (lane 10), or Octa-C6 (lane 11). Gel images were sharpened and brightness and contrast were enhanced to enable all bands to be discerned.

The DNA unwinding assay is sensitive to topoisomerase inhibition and is a measure of DNA intercalation potential. While fluoroquinolones such as ciprofloxacin inhibit topoisomerase activity and intercalation into genomic and/or mitochondrial DNA may contribute to antimicrobial activity, the DNA unwinding assay is used here primarily as a measure of relative risk of genotoxicity. The relaxed pBR322 plasmid migrates as at least five separate bands during electrophoresis, representing multiple topoisomers (Fig. 2A, lane 1). Upon treatment with topoisomerase I, the plasmid is concentrated into one or two bands and does not migrate as far, as the preferred topoisomers are produced (Fig. 2A, lane 2). DQC, Quino-bola, Penta-bola, CM2 and, to a slightly lesser extent, Hepta-bola are intercalators and may also interfere with the action of the enzyme (Fig. 2A, lanes 3 to 7). In contrast, the larger-headgroup bolalipid, Octa-bola, has less of an impact and, while it may inhibit the activity of topoisomerase I, its intercalation is reduced if not eliminated (Fig. 2A, lane 8). Reducing the length of the acyl chain linker for the Octa analogues further reduces the intercalation and may mitigate the enzyme inhibition (Fig. 2A, lanes 9 to 11). The supercoiled plasmid is relaxed through the action of topoisomerase I (Fig. 2B, lanes 1 and 2). This effect is not observed when any of DQC, Quino-bola, Penta-bola, CM2, Hepta-bola, or Octa-bola is also present (Fig. 2B, lanes 3 to 8). Only when the acyl linker is shortened in further Octa analogues is some relaxed plasmid observed, though this effect is again impaired (Fig. 2B, lanes 9 to 11).

### Relationship of structure to antimicrobial activity.

The antimicrobial activities of DQC, CM2 and the new bolalipid analogues were tested against a panel of Gram-negative and Gram-positive bacteria, *Candida* spp., and mycobacteria (Tables 1, 2, and 3). As a class, the bolalipids are all more active against Gram-positive species, *Candida* spp., and both Mycobacterium smegmatis mc^2^ 155, and M. tuberculosis Bleupan than against Gram-negative species. CM2 is a little more potent than DQC against the Gram-negative species, *Candida* spp., and mycobacteria, but it outperforms DQC by an order of magnitude or more against the Gram-positive bacteria. Variation in activity across CM2 and the new bolalipids is modest, but Octa-bola is consistently found to be the most potent of the analogues, and both it and Octa-C10 have notably improved activity against Escherichia coli and Acinetobacter baumannii. All the bolalipids have substantial *in vitro* toxicity, with half-maximal effective concentrations (EC_50_) ranging from only 3.5 μg/mL to 34.7 μg/mL against either HEK293 or HeLa cells for bolalipids with 12- or 10-carbon acyl linkers.

**TABLE 1:** MICs of bolaamphiphiles against a panel of Gram-positive and Gram-negative bacteria, *Candida* spp., and mycobacteria

Category and organism	MIC (μg/ml) of^*a*^:									
	12-bis-THA Cl_2_	Dequalinium	Quino-bola	Penta-bola	Hepta-bola	Octa-bola	Octa-C10	Octa-C8	Octa-C6	Fluconazole
Gram-negative bacteria										
Klebsiella pneumoniae NCTC 13368	32	>32	32	32	32	32	32	32	>32	
Klebsiella pneumoniae M6	**16**	>32	32	32	**16**	**16**	**16**	32	>32	
Acinetobacter baumannii AYE	16	32	32	32	16	**8**	16	32	>32	
Acinetobacter baumannii ATCC 17978	16	32	16	16	16	**8**	**8**	32	>32	
Pseudomonas aeruginosa PAO1	32	>32	>32	32	32	32	>32	32	>32	
Pseudomonas aeruginosa NCTC 13437	32	>32	>32	>32	32	32	>32	32	>32	
Escherichia coli NCTC 12923	4	32	8–16	16	**2**	**2**	**2**	8	>32	
Gram-positive bacteria										
EMR Staphylococcus aureus 15 NCTC 13616	0.25	8	1	0.5	0.25	**0.125**	0.25	1	16	
EMR Staphylococcus aureus 16 NCTC 13277	**0.125**	4	1	0.5	0.25	**0.125**	0.25	1	16	
VS Enterococcus faecalis NCTC 775	2	32	4	4	1	**0.5**	2	4	32	
VR Enterococcus faecalis NCTC 12201	1	16	2	1	**0.5**	1	1	4	16	
VR Enterococcus faecium NCTC 12204	0.5	8	2	1	0.5	0.5	**0.25**	0.5	16	
Fungi										
C. albicans 3179	**0.25**	0.5	**0.25**	**0.25**	0.5	0.5	0.5	2	8	>128
C. albicans 3281	**0.25**	0.5	**0.25**	**0.25**	0.5	0.5	1	2	8	>128
C. auris temp probe	2	4	2	2	2	**1**	4	4	>8	>128
C. glabrata 8018	**0.25**	0.5	**0.25**	**0.25**	**0.25**	0.5	0.5	2	>8	>128
C. tropicalis 8760	**0.25**	0.5	**0.25**	**0.25**	**0.25**	**0.25**	**0.25**	1	8	>128
C. krusei 3876	**0.5**	1	1	**0.5**	**0.5**	**0.5**	2	4	>8	32
C. parapsilosis 3209	1	4	0.5–2	1	1	**0.5**	2	4	>8	0.25
C. parapsilosis	2	8	2	**1**	2	**1**	4	8	>8	0.5
Mycobacteria										
M. smegmatis mc^2^ 155	0.28	0.68	0.49	**0.10**	ND	0.55	ND	0.19	1.66	
M. tuberculosis Bleupan	0.87	3.51	ND	**0.19**	ND	**0.24**	ND	0.49	0.46	

a Bold indicates the best bolaamphiphile. VR, vancomycin resistant; VS, vancomycin susceptible; ND, not determined.

**TABLE 2:** Toxicity of bolaamphiphiles for two cell lines^*a*^

Cell line	Toxicity (EC_50_ [μg/ml]) of:								
	12-bis-THA Cl_2_	Dequalinium	Quino-bola	Penta-bola	Hepta-bola	Octa-bola	Octa-C10	Octa-C8	Octa-C6
HEK293	5.5 ± 2.0	9.9 ± 3.3	13.6 ± 0.6	7.9 ± 1.2	3.5 ± 1.2	3.8 ± 0.3	7.0 ± 0.5	12.9 ± 4.3	**40.0 ± 15.6**
HeLa	7.1 ± 2.0	34.7 ± 13.9	13.9 ± 9.5	9.5 ± 1.7	4.5 ± 0.7	4.5 ± 0.7	10.7 ± 0.3	27.3 ± 1.3	**64.3 ± 3.6**

a Average ± standard error from 3 independent repeats. Bold indicates the best bolaamphiphile.

**TABLE 3:** Selectivity indices of bolaamphiphiles

Organism	Selectivity of^*a*^:								
	12-bis-THA Cl_2_	Dequalinium	Quino-bola	Penta-bola	Hepta-bola	Octa-bola	Octa-C10	Octa-C8	Octa-C6
Gram-negative bacteria (avg)	0.48	0.69	0.55	0.35	0.44	0.53	**1.07**	0.90	<1.63
Gram-positive bacteria (avg)	19.5	2.65	8.94	10.9	10.4	17.4	**23.9**	18.1	2.94
Fungi (avg)	15.8	26.8	**31.8**	22.3	8.00	8.30	11.6	8.48	<6.52
M. tuberculosis Bleupan	7.24	6.35	ND	45.8	ND	17.3	ND	41.0	**113**

a Ratio of average toxicity (HEK293 and HeLa) to average potency. Bold indicates the best bolaamphiphile. ND, not determined.

Despite this, an *in vitro* selectivity index of 23.9 suggests that Octa-C10 might have potential to be developed as a therapeutic for Gram-positive bacterial infections, and it modestly outperforms DQC against bacteria associated with bacterial vaginosis, i.e., Gram-variable Gardnerella vaginalis and Gram-negative Prevotella bivia (Table 4). In contrast, the *in vitro* data suggest that none of the bolalipids are suitable candidates for development for infections caused by other Gram-negative bacteria, at least when used alone.

**TABLE 4:** MICs of DQC and Octa-C10 against a panel of *G. vaginalis* isolates and *P. bivia* NCTC 11156

Organism	MIC (μg/mL)^*a*^	
	DQC	Octa-C10
Prevotella bivia NCTC 11156	2	0.125
*G. vaginalis* NCTC 10287	2	0.5–1
*G. vaginalis* KC1	2	0.5–1
*G. vaginalis* KC2	4	2
*G. vaginalis* KC3	2–4	0.5

a Selectivity indices (ratio of average toxicity [HEK293 and HeLa] to average potency) were 8.58 and 10.7 for DQC and Octa-C10, respectively.

In contrast to Gram-positive bacteria, where the greater *in vitro* selectivity indices are found for bolalipids with larger headgroups, the most attractive *in vitro* selectivity indices for *Candida* spp. are found for bolalipids with the smaller headgroups, with Quino-bola being only marginally better than DQC. The *in vitro* toxicity of the Octa analogues decreases as the acyl linker is shortened, and since the potency against M. tuberculosis Bleupan both is high and does not vary substantially across the tested bolalipids, Octa-C6 is found to have an attractive *in vitro* selectivity index with 2 orders of magnitude difference between active and toxic concentrations.

**FIG 3:**
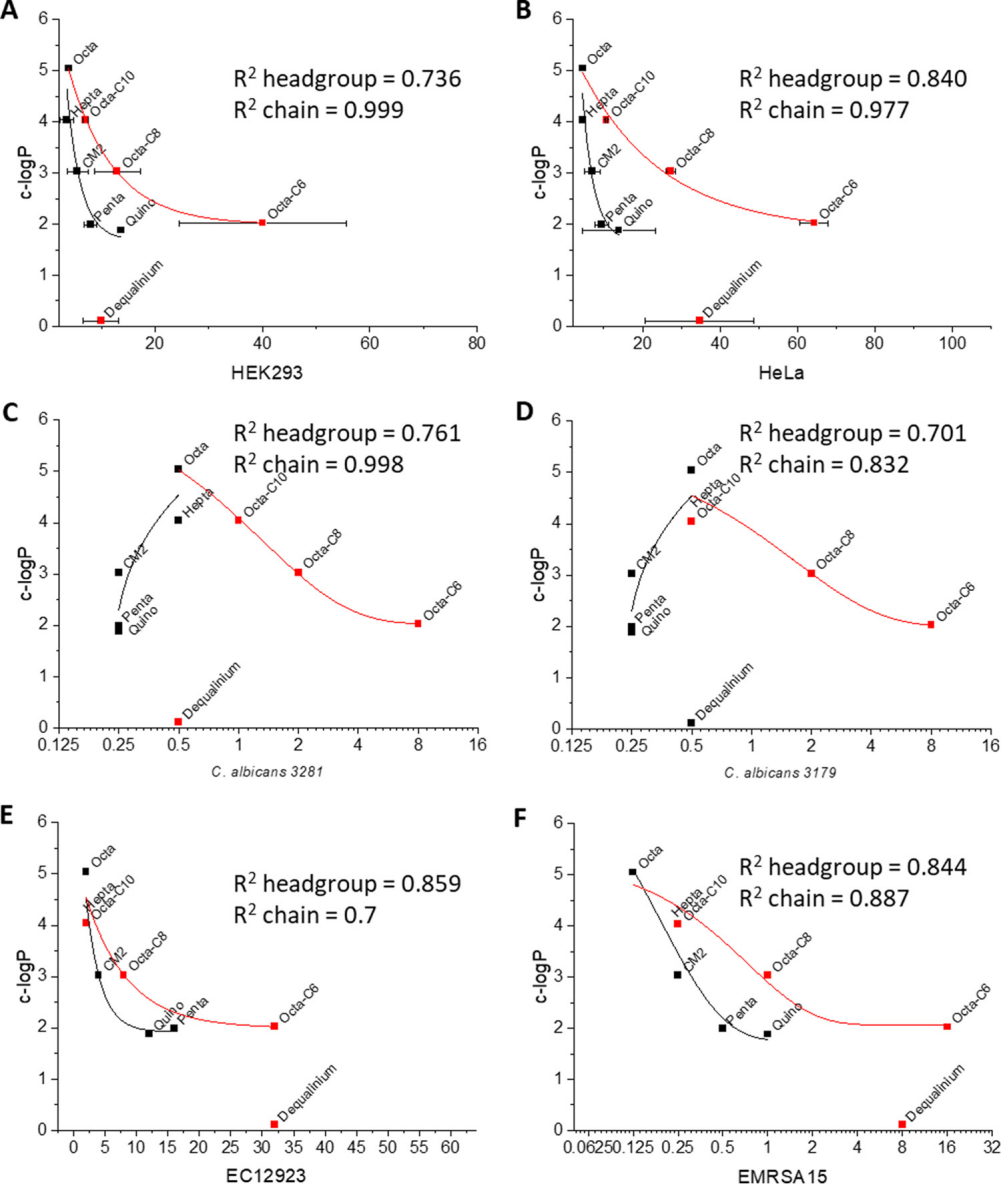
Bolaamphiphiles have a distinct mechanism when targeting fungal pathogens. Toxicity for mammalian cells (EC_50_) (A and B), MICs against C. albicans (C and D) or representative Gram-negative (E. coli 12923) (E) and Gram-positive (EMRSA-15) bacteria (F) (all in micrograms per milliliter) were plotted as a function of calculated logP. Exponential decay fits were used for all headgroup and chain SARs except for the headgroup for C. albicans.

The very different distribution of selectivity indices, according to the target microbial species, highlights the fact that the antimicrobial activity against Gram-negative and Gram-positive bacteria, *Candida* spp., and mycobacteria is underpinned by different properties. To explore this further and consider the role of hydrophobicity, which is altered as a result of the modifications in the SAR, the toxicity to mammalian cells (Fig. 3A and B), potency against Candida albicans (Fig. 3C and D), and representative Gram-negative (Fig. 3E) or Gram-positive (Fig. 3F) bacteria were plotted as a function of the calculated partition coefficient (c-logP).

For the Octa- series, in all cases, both the toxicity and antimicrobial potency decreased as the acyl linker was shortened and the hydrophobicity and partition coefficient decreased (Fig. 3A to F). Similarly, if headgroup modifications alone are considered (for twelve-carbon bolalipids), as the hydrophobicity and partition coefficient decrease, so does the toxicity to mammalian cells (Fig. 3A and B) and potency against bacteria (Fig. 3E and F). In contrast however, the opposite is true of potency toward C. albicans (Fig. 3C and D). Here, bolalipids with a lower hydrophobicity and partition coefficient are more potent, and these are also the bolalipids that are strong intercalators per the unwinding assay discussed above. Notably, we could detect accumulation of Penta-bola in Candida auris TGD1912 at a concentration more than 50 times the MIC without any evidence of plasma membrane damage (see Fig. S1 in the supplemental material); similar behavior for CM2 accumulating in Pseudomonas aeruginosa PAO1 is described below.

10.1128/msphere.00508-22.4 FIG S1 Accumulation of Penta-bola in Candida auris TGD1912. Exponential-phase cells in standard defined medium were incubated with Penta-bola (0.18 mM) for 30 min at 30°C followed by staining with concanavalin A (CF594). Cells were visualized at ×100 magnification via excitation at 358 nm (A) and 580 nm (B) to visualize bolalipid and concanavalin A, respectively; images were then overlaid (C). Fluorescence for Penta-bola is much lower than that for CM2 or Octa-bola, and the cells were challenged with a concentration approximately 54× the MIC of 2 μg/mL to enable detection. Nevertheless, the cells remained intact, indicating that the bolalipid does not act by damaging the plasma membrane and instead accumulates inside the cells. Download FIG S1, PDF file, 0.1 MB. Copyright © 2022 Di Blasio et al. 2022 Di Blasio et al. https://creativecommons.org/licenses/by/4.0/ This content is distributed under the terms of the Creative Commons Attribution 4.0 International license.

### Bolalipids act in synergy with aminoglycosides against P. aeruginosa, with the combinations being more bactericidal.

The antibacterial activity of the bolalipids was investigated in combination with antibiotics relevant to clinical or veterinary practice. In screening, using a fixed (0.25× MIC) concentration of Octa-bola, Octa-C10, or Octa-C6, we found consistent potentiation of both tobramycin and gentamicin antibacterial activities against both sensitive and aminoglycoside-resistant strains of A. baumannii and P. aeruginosa but not E. coli or Klebsiella pneumoniae (Table 5). Using checkerboard assays, we confirmed that strong synergy is observed between the aminoglycoside tobramycin and bolalipids against P. aeruginosa (Table 6). This was observed irrespective of the potency of the bolalipid alone; synergy was observed for DQC, Quino-bola, and Octa-C6, which all have relatively low antibacterial activities. Again, strong synergy was also observed for P. aeruginosa isolates that either have reduced sensitivity to tobramycin (RP73) or are resistant (NCTC 13437). However, the synergy observed between bolalipids and aminoglycosides against A. baumannii and P. aeruginosa does not extend to other species or other antibiotics (Tables S1 and S2).

**TABLE 5:** Synergy between bolalipids and aminoglycosides for two Gram-negative species

Strain	MIC (μg/mL) of^*a*^:							
	Tobramycin				Gentamicin			
	No bola	Octa-Bola	Octa-C10	Octa-C6	No bola	Octa-Bola	Octa-C10	Octa-C6
K. pneumoniae NCTC 13368	8	4	8	16	16	8	16	16
K. pneumoniae M6	1	0.5	**0.25**	1	1	1	1	1
A. baumannii AYE	64	**8**	**4**	**16**	256	**0.5**	**4**	**32**
A. baumannii ATCC 17978	2	**0.125**	**0.5**	**0.5**	2	**0.25**	1	1
P. aeruginosa PAO1	1	0.5	**0.25**	0.5	2	**0.5**	**0.25**	**0.25**
P. aeruginosa NCTC 13437	64	**8**	**4**	**8**	256	**8**	**8**	**8**
E. coli NCTC 12923	2	1	1	2	2	2	1	2

a Broth microdilution assay results for a panel of Gram-negative bacterial strains challenged with antibiotic in the presence of fixed concentrations (0.25× MIC) of the indicated bolalipids. Values in bold represent a ≥2-dilution increase in aminoglycoside potency and hence an FIC of ≤0.5, considered to indicate strong synergy.

**TABLE 6:** Tobramycin acts in synergy with bolalipids against P. aeruginosa^*a*^

Combination	Strain	MIC (μg/mL)				FIC
		Alone		In combination		
		Bolalipid	Antibiotic	Bolalipid	Antibiotic	
DQC-tobramycin	P. aeruginosa PAO1	>32	0.5	8	0.25	<0.75
Quino-bola–tobramycin	P. aeruginosa PAO1	>32	0.5	8	0.0625	<0.375
Penta-bola–tobramycin	P. aeruginosa PAO1	32	0.5	8	0.0625	0.375
CM2-tobramycin	P. aeruginosa PAO1	32	0.5	4	0.0625	0.25
Hepta-bola–tobramycin	P. aeruginosa PAO1	32	0.5	8	0.125	0.5
Octa-bola–tobramycin	P. aeruginosa PAO1	32	0.5	4	0.125	0.375
Octa-C10–tobramycin	P. aeruginosa PAO1	64	0.5	16	0.125	0.5
	P. aeruginosa RP73	8	2	1	0.25	0.25
	P. aeruginosa NCTC 13437	32	64	8	4	0.3125
Octa-C6–tobramycin	P. aeruginosa RP73	64	2	4	0.125	0.0625

a Concordant results from two or more independently repeated experiments are shown.

10.1128/msphere.00508-22.1 TABLE S1 No synergy for Octa-bola or Octa-C10 with other antibiotics against P. aeruginosa. MICs from broth microdilution experiments for two P. aeruginosa strains in the presence or absence of fixed concentrations (0.25× MIC) of the indicated bolalipids. Concordant results or the range of discordant values from two or more independently repeated experiments are shown. Values in bold and underlined represent a ≥2-dilution change in antibiotic potency and hence an FIC of ≥4.0, considered to indicate antagonism. Download Table S1, PDF file, 0.1 MB. Copyright © 2022 Di Blasio et al. 2022 Di Blasio et al. https://creativecommons.org/licenses/by/4.0/ This content is distributed under the terms of the Creative Commons Attribution 4.0 International license.

10.1128/msphere.00508-22.2 TABLE S2 Modest synergy for Octa-C10 with streptomycin or enrofloxacin against *Ac. pleuropneumoniae*. MICs and FIC of bolalipids in combination with either streptomycin or enrofloxacin against a panel of *Ac. pleuropneumoniae* isolates. Concordant results or the range of discordant values from two or more independently repeated experiments are shown. Download Table S2, PDF file, 0.1 MB. Copyright © 2022 Di Blasio et al. 2022 Di Blasio et al. https://creativecommons.org/licenses/by/4.0/ This content is distributed under the terms of the Creative Commons Attribution 4.0 International license.

There was no synergy between Octa-bola or Octa-C10 and a range of antibiotics against either antibiotic-sensitive or -resistant P. aeruginosa. As has been observed previously for DQC, which induces expression of the efflux pump MexCD-OprJ (12), antagonism is consistently observed between both Octa-bola and Octa-C10 and ciprofloxacin, a fluoroquinolone which acts by inhibiting DNA topoisomerases (Table S1). Synergy between Octa-C10 and streptomycin against Actinobacillus pleuropneumoniae isolates was modest (Table S2). In addition, while modest synergy was observed between Octa-C10 and enrofloxacin against some *Ac. pleuropneumoniae* isolates (Fig. S2), there was no effect of combining Octa-C10 and amoxicillin. Synergy was also tested between Octa-C10 and vancomycin, clindamycin, or linezolid against epidemic methicillin-resistant Staphylococcus aureus 15 (EMRSA-15), where the effect was additive, and there was no effect of combining Octa-C10 and rifampin against EMRSA-15.

10.1128/msphere.00508-22.5 FIG S2 Patch-clamp measurements reveal conductance achieved in DPhPG bilayers only at very high bolalipid concentrations. (A to D) Representative traces of membrane activity and all point histograms for 90 μM CM2 (A and B) or DQC (C and D). (E) Time between bolaamphiphile addition and the first appearance of membrane activity in DPhPG membranes. (F) Duration of activity until the membrane was broken (*n* = 4). There was no significant difference between latency and duration with CM2 and DQC. Download FIG S2, PDF file, 0.3 MB. Copyright © 2022 Di Blasio et al. 2022 Di Blasio et al. https://creativecommons.org/licenses/by/4.0/ This content is distributed under the terms of the Creative Commons Attribution 4.0 International license.

**FIG 4:**
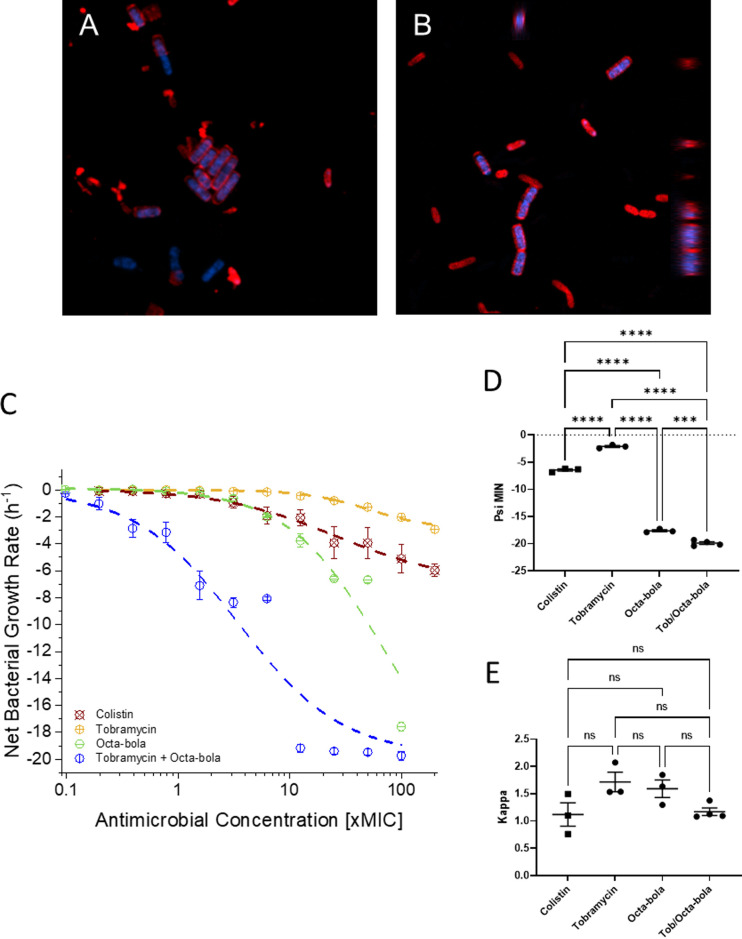
Synergistic activities of bolalipids with tobramycin against P. aeruginosa. Penetration of CM2 into P. aeruginosa PAO1. Bacteria were challenged with 180 μM CM2 in the absence (A) or (B) presence of tobramycin. Red staining represents WGA 555 (wheat germ agglutinin bound to tetramethylrhodamine), while the intrinsic fluorescence of CM2 appears blue. Images were taken 75 min postadministration. (C to E) Pharmacodynamic response of P. aeruginosa RP73 challenged with increasing concentrations of colistin, tobramycin, Octa-bola, or a 2:1 (wt/wt) combination of tobramycin and Octa-bola. The curves are fits of averages from three independent repeated experiments (C). One-way ANOVA with Tukey’s *post hoc* test multiple comparisons for psi-min (D) and kappa (E) highlights the differences in bactericidal rate but not cooperativity between the conditions. ns, *P > *0.05; ***, *P < *0.001; ****, *P < *0.0001.

To better understand the potential mechanism of synergy between the bolalipids and tobramycin against P. aeruginosa, we investigated the ability of DQC and CM2 to damage models of bacterial plasma membranes (Fig. S2) and the ability of CM2 to accumulate within bacteria (Fig. 4A and B). We have developed a patch-clamp approach to study the concentration-dependent effect of antimicrobial peptide (AMP) challenge on models of bacterial plasma membranes (13–15). In this approach, we identify the lowest antimicrobial concentration that induces conductance and then study the membrane activity at this concentration. For AMPs that are supposed to have a predominantly membrane disruptive mechanisms of activity, it is common for conductance to be induced at concentrations between 5 and 10 μM (13, 14), and sometimes as low as 2.5 μM (15). In contrast, 90 μM DQC or CM2 is required to induce any conductance in bilayers composed of DiPhytanoyl PhosphatidylGlycerol (DPhPG) (Fig. S2).

The red-shifted fluorescence emission spectrum of CM2 relative to DQC (Fig. S3) enables its detection by fluorescence microscopy using a filter commonly used to detect Hoechst 33258. When P. aeruginosa PAO1 was challenged with 180 μM CM2, i.e., twice the concentration that induces conductance in the patch-clamp experiments, the bacteria appeared intact and the bolalipid was concentrated within the cells (Fig. 4A). The same behavior was observed when the same treatment was performed in the presence of subinhibitory concentrations of tobramycin (Fig. 4B). Next, we compared the *in vitro* pharmacodynamic behavior of tobramycin alone with that observed when a bolalipid was added in combination. Aminoglycosides such as tobramycin are weakly bactericidal, and we investigated whether the substantial synergistic increase in potency, obtained by combining tobramycin with Octa-bola, might be associated with changes in *in vitro* pharmacodynamic properties (Fig. 4). Although, on average, the most synergy between bolalipids and tobramycin is found with a bolalipid/aminoglycoside ratio of 13:1 (wt/wt), we opted to use a ratio of only 2:1 to test whether only a little bolalipid can affect the PD.

10.1128/msphere.00508-22.6 FIG S3 Normalized emission spectra of 0.45 mM DQC and 0.18 mM CM2 in water (excitation at 350 nm). DQC showed almost no fluorescence at 455 nm, and it could not be detected using a Hoechst 33258 filter in fluorescence microscopy experiments. Download FIG S3, PDF file, 0.01 MB. Copyright © 2022 Di Blasio et al. 2022 Di Blasio et al. https://creativecommons.org/licenses/by/4.0/ This content is distributed under the terms of the Creative Commons Attribution 4.0 International license.

The cooperativity of the dose-dependent activity, as characterized by the steepness of the slope in a dose-response curve and the parameter kappa, was unaffected by the addition of the bolalipid, and this parameter was similar under all conditions tested (Fig. 4C and E). As evidenced by the minimum growth rate (ψ_min_), tobramycin and colistin are both weakly bactericidal against P. aeruginosa RP73 (Fig. 4C and D), but the combination of Octa-bola and tobramycin killed at a much higher rate than either colistin or tobramycin alone (*P < *0.0001). Insufficient material precluded assessment of bactericidal activity above 100× MIC (800 μg/mL); however, Octa-bola was also seen to be strongly bactericidal, albeit only at the highest concentration tested. Taken together, these experiments show that the combination of bolalipids and tobramycin increases the overall potency and bactericidal rate against P. aeruginosa. This was achieved without disrupting the bacterial plasma membrane but while bolalipids penetrated the bacteria cytoplasm.

### Shortening the chain of Octa-bola improves *in vitro* selectivity but impairs therapy.

**FIG 5:**
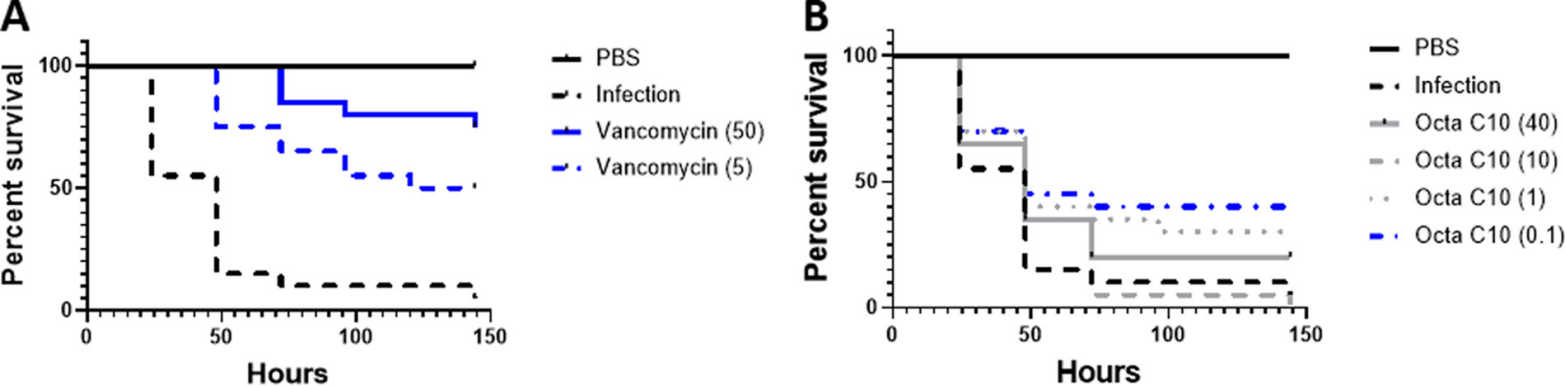
Octa-C10 bolalipid dose determines therapeutic outcomes in an intrahemocoelic G. mellonella model of EMRSA-15 infection. Survival curves for 20 TruLarv larvae are shown for different doses (in milligrams per kilogram) of vancomycin (A) or Octa-C10 (B). Treatment curves that differ (*P < *0.05) with respect to infection only by log rank (Mantel-Cox) tests are shown in blue.

**FIG 6:**
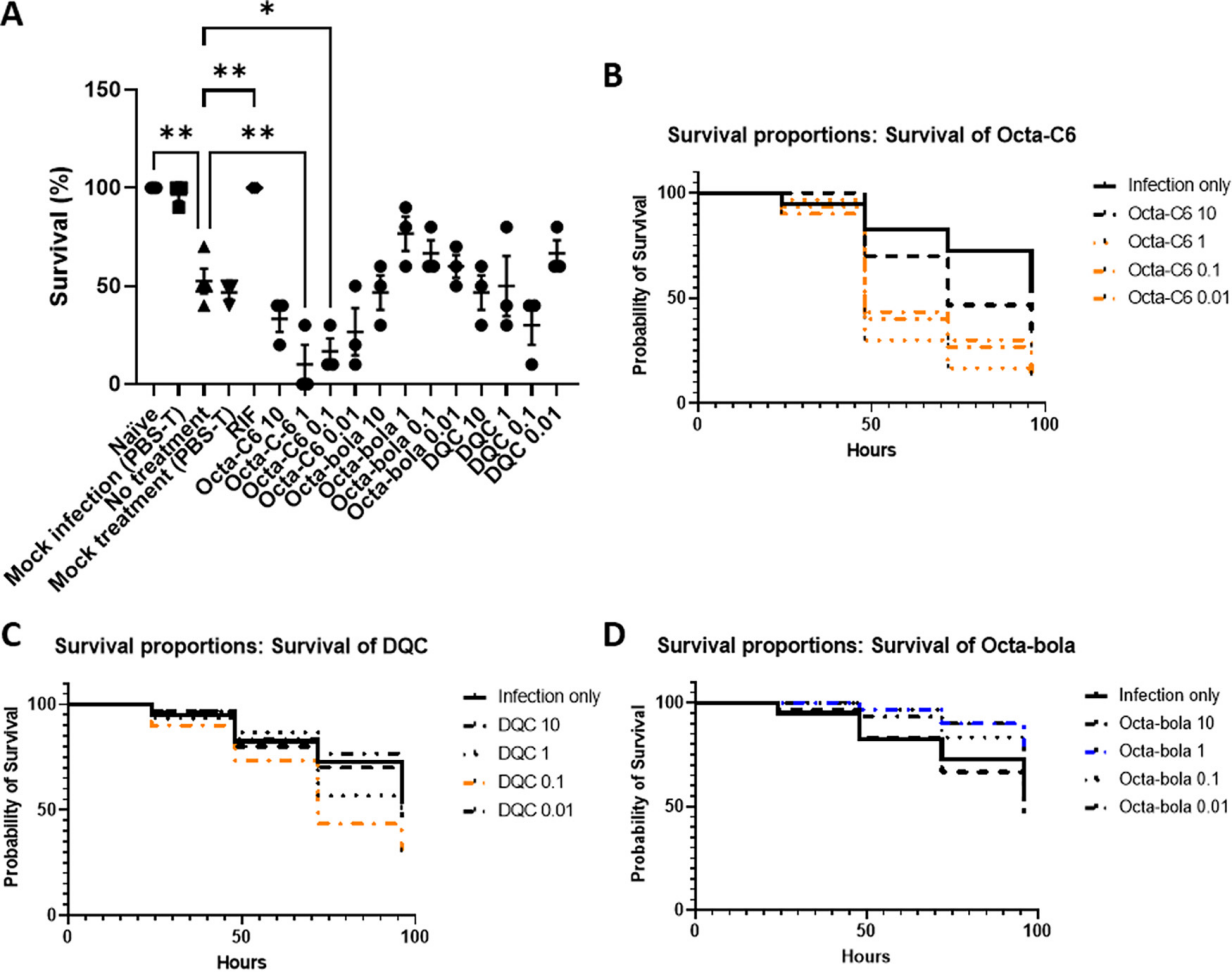
Bolalipid chain length determines therapeutic outcomes in a G. mellonella model of M. tuberculosis Bleupan infection. (A) Survival of 10 TruLarv larvae after 96 h, each treated with the indicated bolalipids (doses are in milligrams per kilogram). Rifampin (RIF; 10 mg/kg) and PBS-T were used as positive and negative controls, respectively. Data were obtained in three independent repeats, and significance is shown relative to the no-treatment value with Dunnett’s correction for multiple comparisons applied. *, *P < *0.05; **, *P < *0.01. (B to D) Survival curves for 30 TruLarv larvae for different doses (in milligrams per kilogram) of Octa-C6 (B), DQC (C), and Octa-bola (D). Curves that differ (*P < *0.05) with respect to infection only by either log rank (Mantel-Cox) or Gehan-Breslow-Wilcoxon tests are shown in orange (exacerbation) or blue (protection).

According to the *in vitro* selectivity indices described above, Octa-C10 and Octa-C6 were selected for evaluation in intrahemocoelic Galleria mellonella models of, respectively, EMRSA-15 (Fig. 5) and M. tuberculosis Bleupan (Fig. 6) infection. G. mellonella responded to treatment of an EMRSA-15 infection with vancomycin in a dose-dependent manner (Fig. 5A). Vancomycin at 50 mg/kg rescued almost 80% of infected G. mellonella larvae (*P < *0.0001), while a tenth of this dose was moderately less effective, rescuing 45% of infected larvae (*P < *0.001). In the same model, the lowest dose of Octa-C10, 0.1 mg/kg, rescued approximately a third of the infected larvae (*P = *0.0167; log rank [Mantel-Cox] test) (Fig. 5B). However, increasing the dose did not lead to further protection, and, indeed, higher doses performed worse, with none of them providing significant protection (Fig. 5B).

All G. mellonella larvae were rescued from M. tuberculosis Bleupan infection by 10 mg/kg rifampin (Fig. 6A). Treatment with Octa-C6, however, did not provide protection, and indeed, survival was significantly worse when the three lower doses (0.01, 0.1, and 1 mg/kg) were administered (Fig. 6B). A dose of 0.1 mg/kg DQC also led to lower survival in this model, though the survival of larvae was indifferent to treatment with higher or lower doses (Fig. 6C). In contrast with the results observed with Octa-C6, treatment with the more hydrophobic Octa-bola did not lead to worse survival and survival improved following treatment with intermediate doses, with 1 mg/kg Octa-bola rescuing about half of the infected larvae (*P* = 0.0316; log rank [Mantel-Cox] test) (Fig. 6D).

To better understand factors that might affect *in vivo* performance, we conducted dynamic light scattering (DLS) measurements to measure the relative propensity of bolalipids to form nanoparticles as an indicator of their tendency to aggregate (Table S3). Of the longer-chain bolalipids, Octa-bola and DQC had the greatest ability to form nanoparticles, followed by CM2 and Penta-bola, with Quino-bola showing the least ability to form nanoparticles. Under no conditions did Octa-C6 give evidence of nanoparticle formation.

10.1128/msphere.00508-22.3 TABLE S3 Dynamic light scattering. DLS measurements are shown for the C_12_ bolalipids and DQC prepared at 0.36 mM, which is the maximum solubility in water of Octa-bola. A derived count rate above approximately 100 kcps is indicative of nanoparticle formation. Octa-bola, DQC, and, to some extent, CM2 and Penta-bola readily form nanoparticles at this concentration, but Hepta-bola and particularly Quino-bola may not. The derived count rate for Octa-C6 does not exceed 26.3 at concentrations up to 1.65 mM, indicating that it will not form nanoparticles at all. Download Table S3, PDF file, 0.1 MB. Copyright © 2022 Di Blasio et al. 2022 Di Blasio et al. https://creativecommons.org/licenses/by/4.0/ This content is distributed under the terms of the Creative Commons Attribution 4.0 International license.

Since none of the bolalipids were toxic to G. mellonella in the absence of infection, we conclude that, in the M. tuberculosis Bleupan infection model and possibly in the EMRSA-15 infection model, bolalipids can act to exacerbate the infection. Since this is not linearly related to dose and the greatest exacerbation was observed for the most soluble bolalipid, Octa-C6, it is apparent that *in vitro* toxicity profiles and selectivity indices may not predict *in vivo* performance.

### Longer-chain bolalipids are effective therapeutics in models of Escherichia coli ATCC 25922 infection.

**FIG 7:**
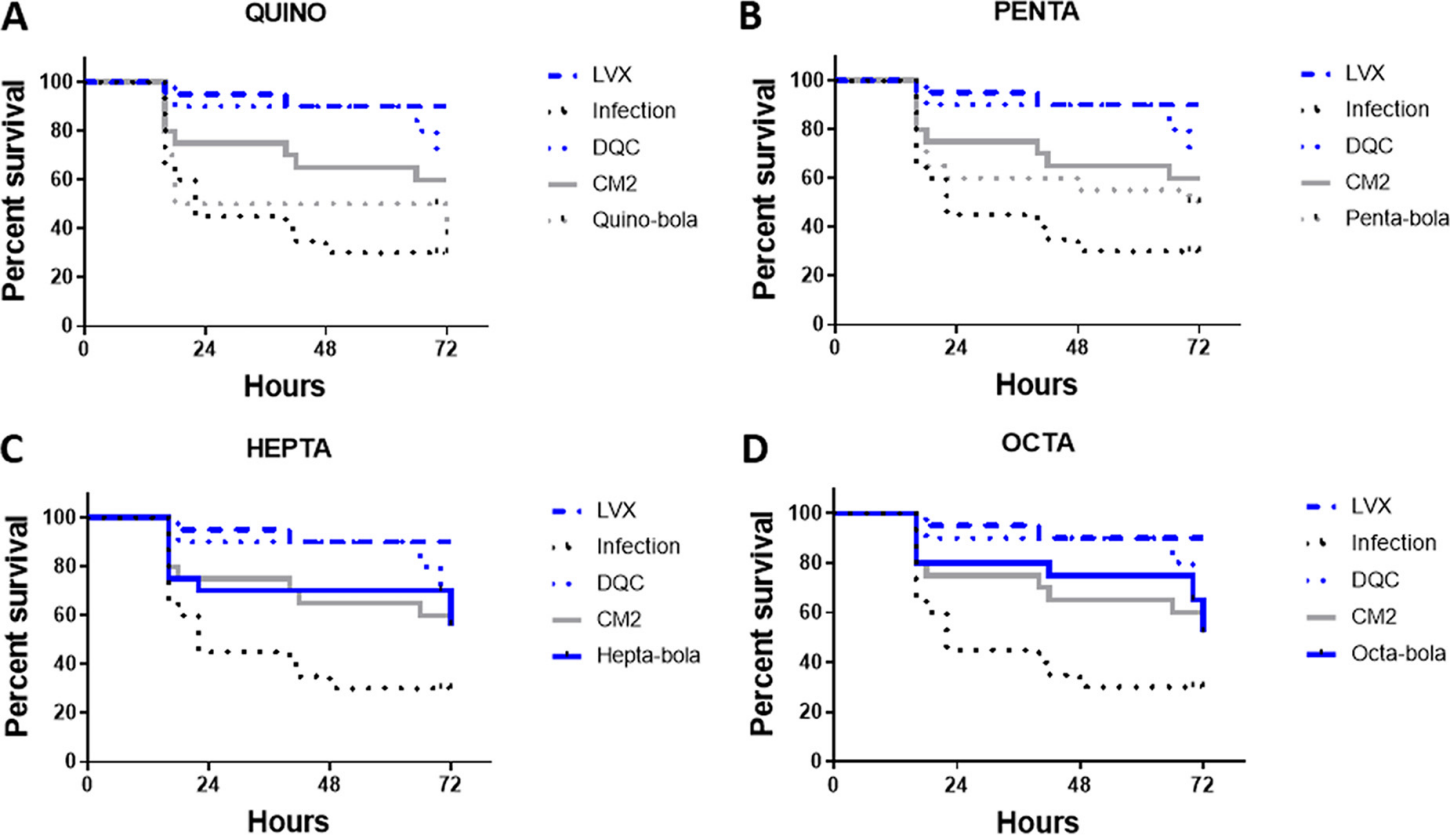
Bolalipids are protective in a G. mellonella model of E. coli ATCC 25922 infection. Survival curves over 72 h are plotted for 20 larvae, each treated with a single 10-mg/kg dose of Quino-bola (A), Penta-bola (B), Hepta-bola (C), or Octa-bola (D), in each case compared with 10 mg/kg CM2 or DQC or 2 mg/kg levofloxacin (LVX). Blue curves indicate significant protection according to a log rank (Mantel-Cox) test (CM2 *P = *0.0527).

Despite the poor *in vitro* toxicity profile and selectivity indices of the twelve-carbon bolalipids, we evaluated whether they may nevertheless provide protection *in vivo* in an intrahemocoelic G. mellonella model of E. coli ATCC 25922 infection. The *in vitro* selectivity indices of the twelve-carbon bolalipids for Gram-negative bacteria are broadly similar, and where antibacterial potency is low, there are corresponding reductions in cytotoxicity, notably for Quino-bola and DQC. Unlike with M. tuberculosis Bleupan, DQC was protective in G. mellonella against E. coli ATCC 25922 infection (Fig. 7). DQC performed significantly better than Quino-bola (*P = *0.041, Gehan-Breslow-Wilcoxon test; *P > *0.05, log rank [Mantel-Cox] test) (Fig. 7A), and neither Quino-bola nor Penta-bola offered significant protection. Other bolalipids, including Hepta-bola (Fig. 7C) and Octa-bola (Fig. 7D), were also significantly protective and were not significantly worse than DQC. The performance of CM2 was between that of the bolalipids with larger and smaller headgroups (Fig. 7), and survival was somewhat better than that obtained in the absence of treatment (*P = *0.0527, log rank [Mantel-Cox] test).

Finally, we assessed whether a combination of Octa-bola and tobramycin or of Octa-C10 and gentamicin might be protective in G. mellonella models of intrahemocoelic Pseudomonas aeruginosa RP73 or Acinetobacter baumannii ATCC 17978 burn wound infections, respectively. G. mellonella larvae are very sensitive to intrahemocoelic infection with P. aeruginosa, and both 1 × 10^2^ CFU and 1 × 10^1^ CFU per larva induced substantial mortality after 96 h (65% and 43%, respectively). Tobramycin at 50 mg/kg rescued 97.5% of the larvae infected with the higher dose (*P < *0.0001, log rank [Mantel-Cox] test), while 5 mg/kg tobramycin rescued 37.5% of the larvae (*P = *0.0084). Addition of 0.25, 1, 4, or 10 mg/kg Octa-bola to 5 mg/kg tobramycin did not improve larva rescue; indeed, the lowest Octa-bola dose impaired the therapy formerly attributed to the tobramycin (*P = *0.0049) but did not exacerbate the infection.

**FIG 8:**
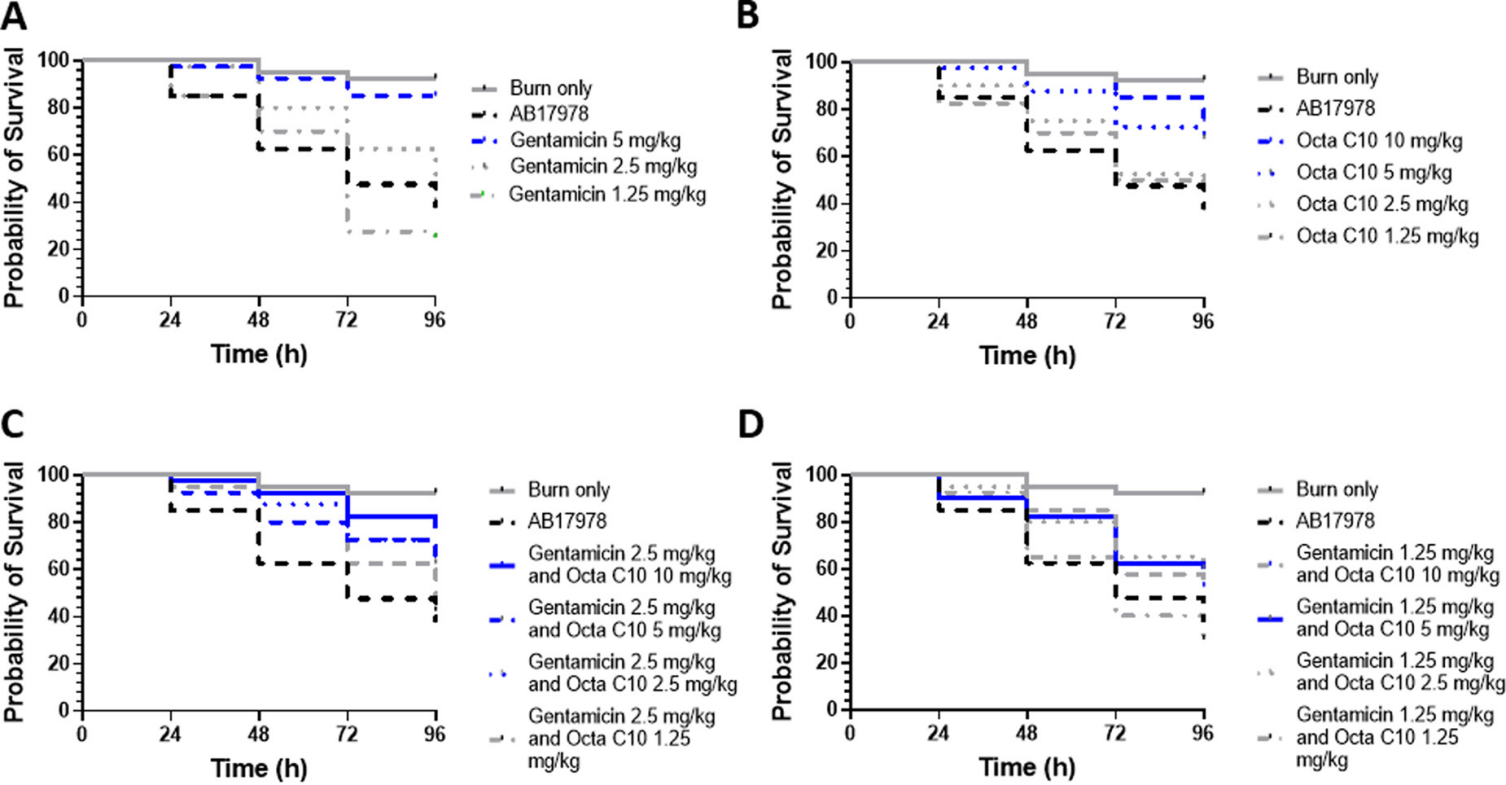
New bolalipids are protective alone or in combination with gentamicin in a G. mellonella model of A. baumannii ATCC 17978 burn wound infection. Survival curves were plotted for 40 larvae, each treated with various doses of gentamicin (A) or Octa-C10 (B) or with 2.5 mg/kg (C) or 1.25 mg/kg (D) gentamicin plus various doses of Octa-C10. Results in each case were compared with burn only or burn plus infection (A. baumannii 17978) over 96 h. Blue curves indicate significant protection (*P < *0.05) against infection, due to therapy, according to a log rank (Mantel-Cox) test (burn-only *P* < 0.0001).

While G. mellonella can tolerate superficial burns, subsequent infection with A. baumannii ATCC 17978 led to 62.5% mortality in 96 h (*P < *0.0001, log rank [Mantel-Cox] test), allowing effective therapy to be quantified (Fig. 8). In a dose-response experiment, 5 mg/kg gentamicin was protective (*P < *0.0001), with 76% larvae rescued, but lower doses (2.5 and 1.25 mg/kg) were ineffective (Fig. 8A). When used alone, Octa-C10 was also protective in this model, with doses of 10 and 5 mg/kg rescuing 60% (*P = *0.0002) and 48% (*P = *0.0042) of the larvae, respectively, but lower doses (2.5 and 1.25 mg/kg) being ineffective (Fig. 8B). When combinations were used to assess *in vivo* synergism, a low dose of gentamicin (2.5 mg/kg) was protective in combination with doses of 2.5 mg/kg Octa-C10 and above (Fig. 3C), rescuing 44% (2.5 mg/kg; *P = *0.0072), 40% (5 mg/kg; *P = *0.0211), and 64% (10 mg/kg *P = *0.0002) in a dose-dependent manner (Fig. 8C). Combinations that contained the lowest dose of gentamicin (1.25 mg/kg) generally fared poorly (Fig. 8D). However, addition of Octa-C10 at 10 mg/kg (*P = *0.0067), 5 mg/kg (*P = *0.0031), or 2.5 mg/kg (*P = *0.0093), a dose that has no effect when applied alone, increased survival relative to 1.25 mg/kg gentamicin alone, where survival was worst (Fig. 8A and D), and the combination with 5 mg/kg Octa-C10 offered modest but significant protection, rescuing 36% (*P = *0.0403). Therefore, the *in vivo* outcomes from attempted burn wound infection therapy indicate that combining gentamicin and Octa-C10 leads to either modest synergy or, at worst at suboptimal doses, an additive effect.

## DISCUSSION

The present SAR study of bolalipids based on CM2 shows that increasing headgroup bulk and/or reducing overall hydrophobicity reduces plasmid intercalation and/or topoisomerase I inhibition, effects expected to mitigate the risk of genotoxicity (16). These modifications also reduced antimicrobial activity against *Candida* spp. In contrast, while reducing the overall hydrophobicity similarly mitigated cytotoxicity and reduced antibacterial potency, increasing the headgroup size had the opposite effect.

Synergy between bolalipids and antibiotics relevant to clinical or veterinary settings was observed. A substantial increase in the P. aeruginosa killing rate was observed when only a small amount of bolalipid was used in combination with tobramycin, which is otherwise weakly bactericidal. However, while synergy was strong for any bolalipid combination with either gentamicin or tobramycin against A. baumannii or P. aeruginosa, no synergy was observed for other Gram-negative bacteria, and it was weaker for another aminoglycoside, streptomycin, against *Ac. pleuropneumoniae*.

The *in vitro* selectivity indices suggested that shorter acyl linker chain bolalipids had the greatest potential for treatment of mycobacteria infections. However, therapy of M. tuberculosis Bleupan infection was achieved only with a bolalipid with a longer acyl linker, while the more soluble analogue, with a shorter acyl linker, exacerbated the infection. Longer acyl linker analogues also offered some protection against E. coli ATCC 25922 despite comparatively poor *in vitro* selectivity indices for Gram-negative bacteria. These findings suggest that the power of *in vitro* assays to predict therapeutic success may be limited for this class of antimicrobial.

We therefore considered the implications of these findings for our understanding of the mechanisms of the bolalipid antimicrobial activity against diverse pathogens and whether and how the bolalipids might be further developed toward use in a broader range of indications.

### Mechanisms of toxicity and antimicrobial activity.

Bolalipid antimicrobials, for which the lead of the class is DQC, have a wide variety of effects on different cell components, and there is no single well-defined target. The mitochondrial toxicity of DQC, which is associated with mitochondrial swelling and inhibition of the mitochondrial respiratory chain, is due to lipophilicity and to its delocalized positive charge, characteristics that enable the molecule to readily penetrate the lipid bilayers of mitochondria (7). DQC also induces selective depletion of mitochondrial DNA in human cells (17) and breaks down mitochondrial DNA in the yeast Saccharomyces cerevisiae, which induces the formation of petite (respiration-deficient) mutants (18). As such, bolalipids may have acute toxic effects on mitochondria and mammalian cells but may also cause delayed-onset effects that nevertheless inhibit both mammalian and fungal cell growth. Consistent with this, since the overall lipophilicity is similar and the delocalized positive charge is retained, none of the headgroup modifications presented here substantially affect cytotoxicity. The present SAR study is also consistent with an important role for DNA binding in inhibiting fungal growth. While all bolalipids retain antifungal activity, likely due to the acute impact on mitochondrial respiration, bolalipids with larger headgroups, which are less able to intercalate in DNA, are also less effective inhibitors of *Candida* spp.

DQC is also rapidly taken up by bacterial cells, penetrating rapidly into the cytoplasm, but its capacity for damaging the plasma membrane is low (19). On entering the bacterial cell, DQC is liable to precipitate cytoplasmic material, most notably nucleic acid, while dehydrogenase activity is also inhibited, potentially impacting respiration (19). In contrast with the antifungal SAR described above, the extent to which nucleic acid precipitation contributes to bolalipid bactericidal activity is unclear, even if this is unrelated to DNA intercalation, since some bolalipids that have the lowest DNA intercalation potential are nevertheless potent antibacterial compounds. Indeed, unlike the results presented above, where bolalipids with larger headgroups were less effective against *Candida* spp., bolalipids with larger headgroups are more potent against both Gram-positive and Gram-negative bacteria *in vitro* and are protective against E. coli infection *in vivo*. This finding is consistent with previous work that showed that delocalized positive charge, larger headgroup, and longer chain are important features for the antimicrobial activity of quaternary ammonium surfactants (20). This study also identified three modes of antibacterial action in the series of quaternary ammonium surfactants. At low concentrations, bacterial energetics and cell division were impaired. At intermediate concentrations membrane permeabilization and inhibition of electron transport was observed. Disruption of bacterial membranes and cell lysis occurred only at concentrations close to the critical micellar concentration. Given the relatively high potency of the bolalipids described here, the structural modifications that impact antibacterial potency may therefore modulate bolalipid impact on bacterial energetics and/or cell division.

### Mechanism of bolalipid-aminoglycoside synergy.

The ability of the cationic bolalipids to accumulate in cells may also underpin their ability to act in strong synergy with the aminoglycosides tobramycin and gentamicin against P. aeruginosa (and A. baumannii). Tobramycin is positively charged at physiological pH and interacts electrostatically with bacterial membranes but requires entry to the bacterial cytosol to exert its main mechanism of action, which consists of inhibiting bacterial protein synthesis by preventing the formation of the complex between the bacterial 70S ribosome and mRNA. Being a very hydrophilic molecule, however, it cannot passively cross the membrane and instead exploits the proton motive force to enter the cell (21).

An antimicrobial that acts in synergy with tobramycin might therefore be expected to facilitate its transfer to ribosomes after crossing the cytoplasmic membrane, interfere with aminoglycoside-modifying enzymes, or simply increase its intracellular concentration. It appears unlikely that the latter is achieved by bolalipids damaging the cell wall and hence increasing permeability, since their potential to disrupt bilayer integrity was shown here to be low. Further, the same potentiation of tobramycin was obtained even with bolalipids (e.g., Octa-C6) that are relatively inactive against bacteria when used alone, and much weaker or no synergy was observed between bolalipids and either tobramycin or gentamicin against other Gram-negative bacteria. Instead, it is possible that further investigation of the phenomenon will reveal that increased tobramycin intracellular accumulation is achieved by stimulating its uptake. This has been observed for amphiphilic rhamnolipids, produced by P. aeruginosa, which facilitate proton motive force-independent tobramycin uptake in Staphylococcus aureus, and fumarate, which increases cellular respiration and proton motive force (22, 23). If the synergistic combination of bolalipids and tobramycin relies on bolalipids increasing P. aeruginosa cellular respiration, then the infection setting may strongly influence whether this is possible and the scope for the future development of such combinations in differing indications. Nevertheless, as recently achieved by others using photodynamic therapy (24) or light-activated molecular machines (25), successful therapy of A. baumannii-infected burn wounds in G. mellonella, with Octa-C10 both alone and in combination with gentamicin, suggests that further evaluation in mammalian infection models may be warranted. While gentamicin sulfate cream is widely used to treat burn wound infections, aminoglycoside resistance in P. aeruginosa and A. baumannii is an acute problem (26, 27). Future research may uncover whether bolalipids can be used to counter aminoglycoside resistance in burn wounds and, due to the rapid bactericidal activity, treat infections more rapidly.

### Factors that will influence further development.

None of the bolalipid headgroup or acyl linker modifications substantially affect antimicrobial activity against mycobacteria. As such, a promising *in vitro* selectivity index for M. tuberculosis Bleupan activity over toxicity was obtained for Octa-C6, a bolalipid that also has weak intercalation/topoisomerase I inhibition potential. However, rather than offering protection against infection in G. mellonella, at relatively low doses Octa-C6 exacerbated the infection. Octa-bola, which was similarly potent *in vitro* but which had a much poorer selectivity index, was paradoxically much better tolerated *in vivo* and offered protection at intermediate doses. It was also notable that DQC also exacerbated M. tuberculosis Bleupan infection in G. mellonella. DQC was substantially less potent than Octa-bola against M. tuberculosis Bleupan *in vitro* but was nevertheless more potent against this pathogen than it was against E. coli, against which DQC offered some protection *in vivo*. A better understanding of the relationships between *in vitro* activities and *in vivo* therapeutic performance may require investigation of how different classes of antimicrobials interact with, and different pathogens respond to, the innate immune system. Notably, antimicrobials that are mitochondrial poisons will likely impair the oxidative burst of hemocytes. However, the relative *in vivo* performance against M. tuberculosis Bleupan does suggest a pathway for future development. CM2 was designed to have better self-assembly properties than DQC, with acyl linker length and bulkiness of the headgroup aliphatic residue being important determinants, and the headgroup of Octa-bola is bulkier still. As expected, Octa-bola had the greatest ability to form nanoparticles and Octa-C6 the least, reflecting substantially different tendencies of these bolalipids to form aggregates. These data are insufficient to provide a mechanistic explanation but suggest future avenues to improve understanding of determinants of *in vivo* outcomes. We speculate that self-assembly, or effects related to this property, may have a critical impact on parameters such as biodistribution and local versus generalized impacts on the host innate immune system. Therefore, in spite of poor *in vitro* toxicity profiles, better self-assembly properties may be critical in determining *in vivo* therapeutic outcomes, and bolalipids with this property may offer a better foundation for future development.

### Conclusion.

Taken together, the results of the SAR approach used in the present study provide a better understanding of the differing mechanisms that underpin bolalipid antimicrobial activity against Gram-positive and Gram-negative bacteria, *Candida* spp., and mycobacteria. Further, while the genotoxicity of the bolalipids may be mitigated by structural modification, cytotoxicity is unaffected and remains a substantial hurdle to repurposing bolalipids for a wider range of microbial indications. Nevertheless, the discovery of bolalipid synergy with aminoglycosides and resensitization of aminoglycoside-resistant strains of P. aeruginosa and A. baumannii, coupled with the observation of successful therapy in four of five G. mellonella infection models with bolalipids with longer acyl linkers, suggests that formulations that can serve to mitigate the availability of soluble bolalipid before reaching the microbial target may unlock wider utility of this group of antimicrobial lipids, even if it is initially limited to topical application.

## MATERIALS AND METHODS

### Synthesis.

The designed compounds (Fig. 1) were synthesized via solution chemistry techniques. Purification procedures involved flash chromatography, liquid-liquid extraction, high-pressure liquid chromatography (HPLC), and ion exchange. All the intermediates and the final products were characterized by ^1^H and ^13^C nuclear magnetic resonance (NMR), low-resolution mass spectrometry (LRMS), and high-resolution MS (HRMS) techniques to verify and confirm the formation of the desired compounds. Details about the synthesis and purification processes can be found in the supplemental material.

### Unwinding assay.

A DNA unwinding assay kit (Inspiralis Limited, Norwich, UK) consists of wheat germ topoisomerase I assay buffer (50 mM Tris HCl [pH 7.9], 1 mM EDTA, 1 mM dithiothreitol [DTT], 20% [vol/vol] glycerol, 50 mM NaCl) dilution buffer (50 mM Tris HCl [pH 7.9], 1 mM EDTA, 1 mM DTT, 50% [vol/vol] glycerol, 500 mM NaCl), wheat germ topoisomerase I (5 U/μL), supercoiled plasmid pBR322 (1 μg/μL), and relaxed plasmid pBR322 (1 μg/μL). For the experiments, 2× DNA loading buffer containing 40% (wt/vol) sucrose, 100 mM Tris-HCl (pH 8), 1 mM EDTA, and 0.5 mg/mL bromophenol blue was prepared. The enzyme was diluted 1/5 with dilution buffer before being used in the assay. For the assay, 3 mM stock solutions in methanol of the compounds to be tested were prepared. An appropriate number of 1.5-mL Eppendorf tubes were filled with 25 μL of a mix consisting of assay buffer (15 μL of buffer per sample), supercoiled or relaxed pBR322 (0.5 μg/assay), and ultrapure water. In the first two tubes, 1 μL of methanol was added to test the effect of the solvent on both the plasmid and the enzyme. Subsequently, 1 μL of the test compounds was added to the remaining tubes, and the tubes briefly vortexed and incubated at room temperature for 5 min. After that, 2 μL of dilution buffer was added in the first tube, whereas 2 μL of enzyme diluted solution was added to the remaining tubes. The tubes were gently vortexed and incubated for 30 min at 37°C in a water bath. After this time, the reaction was stopped by adding 20 μL of water and 50 μL of water-saturated butanol to each tube. The tubes were vortexed and centrifuged for 1 min at 14,800 rpm. The lower aqueous layer of each tube was removed and transferred to a new tube containing 30 μL of chloroform-isoamyl alcohol (24/1) and 50 μL of loading buffer. The tubes were briefly vortexed and centrifuged for 1 min. Twenty microliters of the upper layer of each tube was loaded onto a 1% (wt/vol) agarose gel, and the gel was run at 80 V for approximately 4 h in Tris-acetate-EDTA buffer (40 mM Tris acetate and 1 mM EDTA). Subsequently, the gel was stained with ethidium bromide (1 μg/mL) for 15 min, briefly destained (5 to 10 min) in water, and visualized with a Multi-Doc Imaging M-26X UVP transilluminator.

### Antibacterial activity assay.

The antibacterial activity of the bolalipids was assessed through a modified 2-fold broth microdilution assay with modal MICs generated from at least three biological replicate experiments (28). The method broadly followed EUCAST methodology, with non-cation-adjusted Mueller-Hinton broth replacing cation-adjusted Mueller-Hinton broth for panels of Gram-negative bacteria, Gram-positive bacteria, and fungi (Tables 1 to 3), Middlebrook 7H9 broth (Difco, Detroit, MI, USA) supplemented with 10% (vol/vol) oleic acid-albumin-dextrose-catalase (OADC) enrichment (Becton, Dickinson, Franklin Lakes, NJ, USA) and 0.5% (vol/vol) glycerol for Mycobacterium smegmatis mc^2^ 155 and M. tuberculosis Bleupan (Tables 1 to 3), brain heart infusion (BHI) broth with 5% horse serum for Gardnerella vaginalis and Prevotella bivia (Table 4), and BHI with 5% fetal bovine serum (FBS) and 0.01% NAD for Actinobacillus pleuropneumoniae and Glaesserella parasuis (Table 5). *G. vaginalis* and *P. bivia* MIC testing was conducted under anaerobic conditions generated using Thermo Scientific Oxoid AnaeroGen. Bolalipids and antibiotics were diluted in a 2-fold dilution in media down a sterile, polypropylene 96-well plate (Greiner Bio-One GmbH, Frickenhausen, Germany). Bacteria were then added, back-diluted from an overnight culture, at a starting concentration of 5 × 10^5^ CFU/mL. Plates were incubated statically at 37°C for 20 h, and the optical density at 600 nm (OD_600_) was determined using a Clariostar plate reader (BMG Labtech). The MIC was defined as the lowest concentration where growth was <0.1 above the background absorbance. Synergy was measured using either a fixed concentration of bolalipid (0.25× MIC) combined with doubling dilutions of antibiotic, or standard microdilution checkerboard assays under the same conditions as the MICs (29). For the fixed-concentration assays, 2-fold dilution series of the antibiotics were prepared, and then bolalipid was added at a final concentration of 0.25× MIC, followed by addition of bacteria. The MIC of the antibiotic in combination with bolalipid was defined as above. For the checkerboard assays, 2-fold dilution series of each antibiotic were prepared in separate 96-well plates and then combined into one before addition of bacteria. The growth/no-growth interface was determined using the same definition as the MIC. The fractional inhibitory concentration (FIC) was calculated from the most synergistic well on the plate for three independent repeats, and values are presented as averages and standard deviations. The FIC is calculated as (MIC of compound A in combination with B/MIC of compound A alone) + (MIC of compound B in combination with A/MIC of compound B alone). MICs were determined on the same plates as the FICs to increase reproducibility. FIC values of ≤0.5 were considered to indicate strong synergism and, consistent with a recent re-evaluation of FIC, which stressed the importance of also measuring the MIC in the same microarray plate, values of 0.5 to <1 were considered to indicate weak synergism (29).

### Dynamic light scattering.

Stock solutions of bolalipids were prepared by dispersing the powder in ultrapure water, then stirring vigorously by vortexing, and sonicating in a 40°C bath sonicator for 20 min. The concentration of each stock solution corresponds to the highest solubility achieved in water for each bolalipid, using the method described above. DLS experiments were performed on a Malvern Zetasizer Nano (Malvern Instruments, Worcestershire, UK) in back-scattering mode at 20°C. There were three independent measurements of each sample and 12 runs for each measurement, with 10 s taken for each run. The attenuator index and measurement position were automatically adjusted. Fifty microliters of each sample was transferred into polystyrene semi-microcuvettes for determination of UV and visible wavelengths (Sarstedt).

### Electrophysiology (patch-clamp) experiments.

Giant unilamellar vesicles (GUVs) composed of DiPhytanoly PhosphatidylGlycerol (DPhPG) were prepared in the presence of 1 M sorbitol by the electroformation method in an indium-tin oxide (ITO)-coated glass chamber connected to the Nanion Vesicle Prep Pro setup (Nanion Technologies GmbH, Munich, Germany) using a 3-V peak-to-peak AC voltage at a frequency of 5 Hz for 140 min at 37°C (30–32). Bilayers were formed by adding the GUV solution to a buffer containing 250 mM KCl, 50 mM MgCl_2_, and 10 mM HEPES (pH 7.00) onto an aperture in a borosilicate chip (Port-a-Patch; Nanion Technologies) and applying 7,000 to 9,000 Pa negative pressure, resulting in a solvent-free membrane with a resistance in the GΩ range. Diphytanoyl chains were used here for practical reasons, since, unlike lipids with mixed palmitoyl-oleoyl chains, these lipids do not undergo the main, temperature-dependent transition from disordered fluid into the all-*trans* configuration and remain in the same phase between −120° and +120°C (33) while, crucially, the membranes composed of these lipids are mechanically stable and have high specific resistance (34), essential for electrophysiology experiments. After formation, a small amount of bolalipid stock solution (in water) was added to 50 μL of buffer solution in order to obtain its active concentration. All the experiments were carried out with a positive holding potential of 50 mV. The active concentration, the concentration at which the bolalipid first showed membrane activity, for each bolalipid was obtained through a titration performed under the same conditions. For all the experiments, a minimum of 6 concordant repeats were done. Current traces were recorded at a sampling rate of 50 kHz using an EPC-10 amplifier from HEKA Elektronik (Lambrecht, Germany). The system was computer controlled by PatchControl software (Nanion) and GePulse (Michael Pusch, Genoa, Italy [http://users.ge.ibf.cnr.it/pusch/software-mik.htm]). The data were filtered using the built-in Bessel filter of the EPC-10 amplifier at a cutoff frequency of 10 kHz. The experiments were performed at room temperature. Data analysis was performed with the pClamp 10 software package (Axon Instruments).

### Fluorescence spectroscopy.

Bolalipid fluorescence spectra were recorded in water; 0.45 mM and 0.18 mM solutions of CM2 and DQC were prepared by dispersing the powder in ultrapure water, then stirring vigorously by vortexing, and sonicating in a bath sonicator at 40°C. A Varian Cary Eclipse spectrophotometer was used to record fluorescence intensity. Seven hundred microliters of each bolalipid solution was transferred into a Hellma Analytics UV quartz cuvette with a light path of 10 mm and used for each recording. For both bolalipids, the excitation wavelength was set to 350 nm while the emission range was from 352 nm to 600 nm, and the averages for 20 scans were obtained.

### Fluorescence microscopy.

P. aeruginosa PAO1 was incubated with CM2 alone or in combination with tobramycin. A single colony of bacteria was collected from a Luria-Bertani (LB) agar plate and resuspended in 10 mL of LB broth overnight at 37°C, with shaking at 150 rpm. The following morning, a 2% (vol/vol) dilution was done in fresh LB broth, and the bacteria suspension was grown at 37°C with shaking at 150 rpm until it reached an OD_600_ of 0.3. The surface of microscopy SuperFrost glass slides (Thermo Fisher Scientific, Massachusetts, USA) was marked with a PAP pen, in order to design two square confined areas per glass slide, where the bacteria were deposited. One hundred microliters of bacterial suspension was transferred into sterile Eppendorf tubes and incubated with equal volumes of bolalipid suspension for 1 h. The bolalipid final concentration was 0.18 mM. The fluorescent Alexa Fluor 555 conjugate of wheat germ agglutinin (WGA) (Thermo Fisher Scientific) lectin membrane dye (10 μg/mL final concentration) was added to the suspension, and the bacteria were incubated for 45 min more. At the end of incubation, 100 μL was spotted in each half of the SuperFrost glass slides and incubated for 45 min more. Glass slides were then washed three times with phosphate-buffered saline (PBS) and left to dry for 5 min before addition of one drop of Fluoromount (Sigma-Aldrich, Germany) aqueous mounting medium. Confocal images were acquired with a Zeiss LSM800 microscope (Zeiss, Germany) and elaborated with ZEN software. Excitation and emission filter wavelengths were, respectively, 352 nm and 455 nm for CM2 and 541 nm and 565 nm for WGA555.

Candida auris cells were picked from a yeast extract-peptone-dextrose (YPD) agar plate and resuspended in 35 mL of sterile distilled water (dH_2_O). After resuspension, 150 μL of cells was inoculated in 100 mL of YPD broth overnight at 30°C with shaking at 130 rpm and grown to exponential phase (5 × 10^6^ to 5 × 10^7^ cells/mL). Cells (6 × 10^8^) were washed in standard defined (SD) medium twice (pelleted at 1,500 × g for 5 min and resuspended in 10 mL SD medium [yeast nitrogen base without the amino acids d-glucose, methionine, adenine, histidine, tryptophan, uracil, leucine, and lysine]), followed by resuspension in 1 mL SD medium containing 0.18 mM Penta-bola, and incubated at 37°C for 30 min. After treatment with the Penta-bola, cells were pelleted and resuspended in Hanks’ balanced salt solution (HBSS) and 50 μg/mL CF594 concanavalin A (Biotium). After incubation at 37°C for 30 min, cells were washed once with HBSS and resuspended in 1 mL of HBSS. Polysine adhesion slides (Thermo Scientific) were prepared by spreading 20 μL of treated cells and allowing them to dry for 30 min before adding one drop of Fluoromount (Sigma-Aldrich, Germany) aqueous mounting medium. Fluorescence images were acquired using a Leica fluorescence microscope at ×100 magnification. Excitation and emission filter wavelengths were 358 nm and 461 nm for Penta-bola and 593 nm and 614 nm for CF594 concanavalin A, respectively.

*In vitro* PD assays were performed with Pseudomonas aeruginosa RP73 cultured in Roswell Park Memorial Institute 1640 medium (RPMI) supplemented with 5% Mueller-Hinton broth (MHB) (35). Bacteria were cultured overnight in 10 mL of RPMI–5% MHB at 37°C and diluted just prior to plate inoculation to an OD_600_ of 0.002. Stock solutions of colistin or tobramycin were prepared in sterile MilliQ water at a concentration of 200× MIC. Octa-bola was prepared in methanol at 2,000× MIC and diluted in water to 200× MIC. A dilution series was made in the top row of a polypropylene 96-well plate from 200× MIC to 0.2× MIC in a volume of 100 μL, to which 100 μL of the bacterial suspension was added to have a total of 1 × 10^6^ log-phase CFU in 200 μL. The first time zero sample was taken <30 s after addition of bacteria to the plate, with further samples taken at appropriate intervals thereafter. Octa-bola–tobramycin-challenged bacteria were sampled every 20 min for 120 min due to rapid killing, while tobramycin- and colistin-challenged bacteria were sampled every hour for 6 h. Fifteen microliters was removed from each well, diluted 1:1,000 in phosphate-buffered saline, and plated onto RPMI–5% MH agar plates. The plates were incubated at 37°C overnight for CFU counting.

CFU data were log_10_ transformed, and the bacterial net growth rate was determined from the increase or decrease in CFU during the time of exposure to the antibiotics as the coefficient of a linear regression of log_10_ CFU as a function of time. The intercept of the regression was fixed by forcing the regression lines through the first CFU measurement (0 min) at a given antimicrobial concentration. The pharmacodynamic function according to Regoes et al. (36) describes the relationship between bacterial net growth rate (ψ) and the concentration of an antimicrobial (*a*): ψ_max_ − ([ψ_max_ − ψ_min_][*a*/zMIC])^κ^/([*a*/zMIC]^κ^ − [ψ_min_/ψ_max_]). Fitting this function to the net bacterial growth rates in OriginPro 2020 (OriginLab Corporation, Northampton, MA) generates the parameters ψ_min_ and ψ_max_ (minimum and maximum growth rates, respectively), zMIC (the pharmacodynamic MIC), and κ (a measure of the cooperativity). Average parameters obtained from fits of three or more independently repeated experiments were compared by one-way analysis of variance (ANOVA) with Tukey’s *post hoc* test. Since the CFU data are log_10_ transformed, the net growth rates are reported to three significant figures.

### Galleria mellonella infection models.

The antimicrobial activities of DQC, CM2, and its analogues Quino-bola, Penta-bola, Hepta-bola, and Octa-bola against Escherichia coli ATCC 25922 were tested in Galleria mellonella larvae. All assays were performed on two separate occasions using a group size of 10 larvae; the larvae were kept in separate petri dishes and in a static incubator at 37°C during the period of the experiment. A suspension of E. coli ATCC 25922 was prepared by transferring two colonies of E. coli ATCC 25922 from a fresh agar plate into PBS and adjusting the absorbance to a McFarland 0.5 standard. The number of CFU per milliliter of this suspension was determined and corresponded to 1 × 10^8^ CFU/mL. Larvae were injected beneath the tegument with 10 μL of a 1 × 10^7^-CFU/mL bacteria suspension, corresponding to 1 × 10^5^ CFU per larva. A 50-μL Hamilton syringe with a G27 needle was used for the injection. Stock solutions of bolalipid analogues were prepared at a concentration of 250 μg/mL with DQC at 200 μg/mL due to its poor solubility. Ten microliters of stock solution was injected into each larva 1 h after infection (10 mg/kg). Levofloxacin (LVX; 50 μg/mL = 2 mg/kg) and PBS were used as positive and negative controls, respectively. A noninfected PBS injection was included to control for the effects of injection. Survival was monitored over a period of 70 to 72 h. Changes in G. mellonella survival were analyzed by the log rank (Mantel-Cox) method and plotted as Kaplan-Meier survival curves using GraphPad Prism version 7.

The procedure was similar for EMRSA-15, P. aeruginosa RP73, and M. tuberculosis Bleupan infections with the following modifications. For EMRSA-15, larvae were injected with 10 μL of a 1 × 10^7^ CFU/mL suspension with treatment by single injection initiated 2 h postinfection. Survival was monitored for 144 h postinfection. For P. aeruginosa RP73, overnight cultures were prepared from a single colony, and following dilution in PBS, larvae were injected with 10 μL of either a 1 × 10^4^-CFU/mL or 1 × 10^3^-CFU/mL bacterial suspension, corresponding to 1 × 10^2^ CFU or 1 × 10^1^ CFU per larva. Treatment was a single injection, 2 h after infection, with 5 or 50 mg/kg vancomycin as controls. Survival was monitored over a period of 96 h.

For M. tuberculosis Bleupan, a mid-log-phase culture was prepared from a glycerol stock and washed and diluted in PBS containing 0.05% polysorbate-80 (PBS-T) to produce a bacterial suspension of 2 × 10^9^ CFU/mL. Of this, 10 μL was injected, corresponding to 2 × 10^7^ CFU per larva. Treatment was given as a single injection, 1 h postinfection. Larval survival was monitored over 96 h. For positive and negative controls, rifampin (RIF; 200 μg/mL = 10 mg/kg) and PBS-T were used, respectively. A noninfected control using PBS-T was used as described above.

In addition to the intrahemocoelic infection/therapy models described above, a recently described G. mellonella burn wound model was adopted (37). The burn was created using the flat head of a nail, which was heated in a blue Bunsen burner flame until red hot, cooled for 15 s, and superficially applied for 2 s to generate a 2-mm^2^ burn. Larvae were then infected with a single colony of A. baumannii ATCC 17978 applied directly to the burn site, or sterile phosphate-buffered saline was applied as a control. Treatment was applied topically 1 h after infection in a volume of 5 μL. Survival was monitored over 96 h.
