# Supplementary figures and images for: Bolaamphiphile Analogues of 12-bis-THA Cl2 Are Potent Antimicrobial Therapeutics with Distinct Mechanisms of Action against Bacterial, Mycobacterial, and Fungal Pathogens
Source: mSphere. 2022 Dec 13;8(1):e00508-22. doi: 10.1128/msphere.00508-22 (PMC9942557; doi:10.1128/msphere.00508-22)

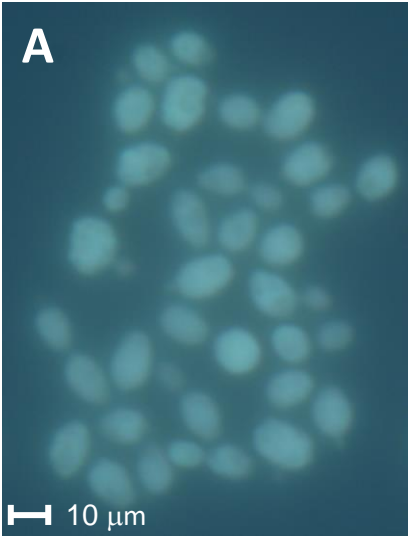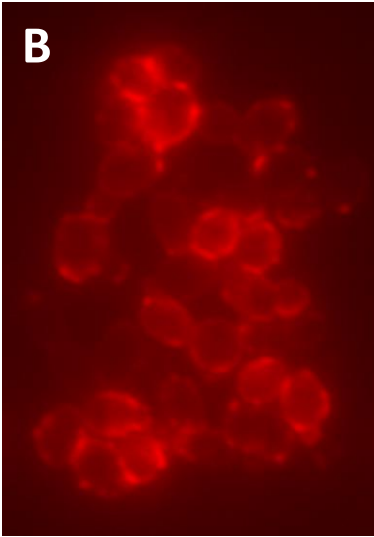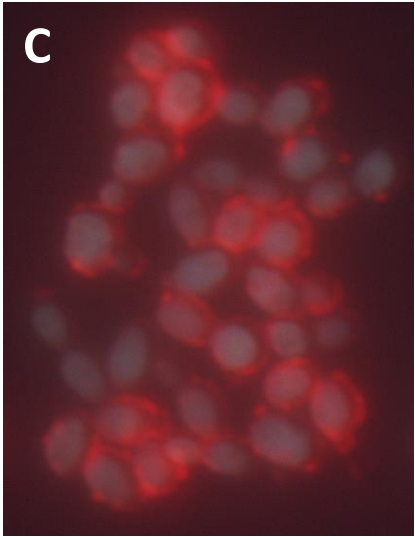

Supplement: FIG S1 [file msphere.00508-22-s0004.pdf]

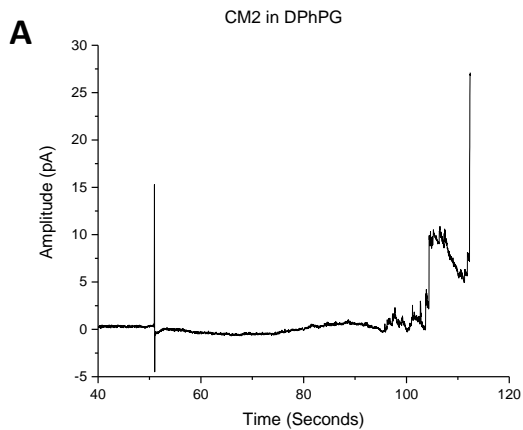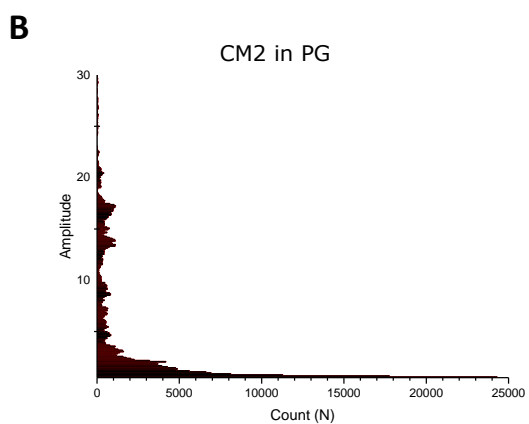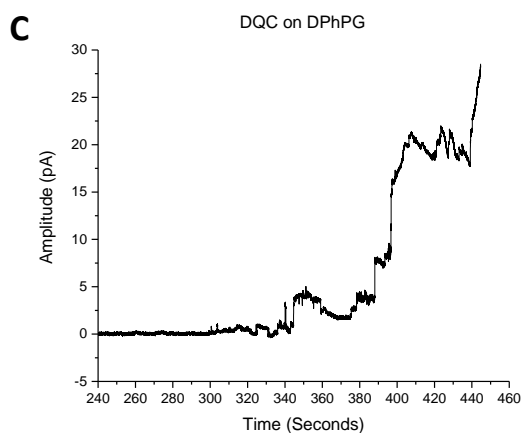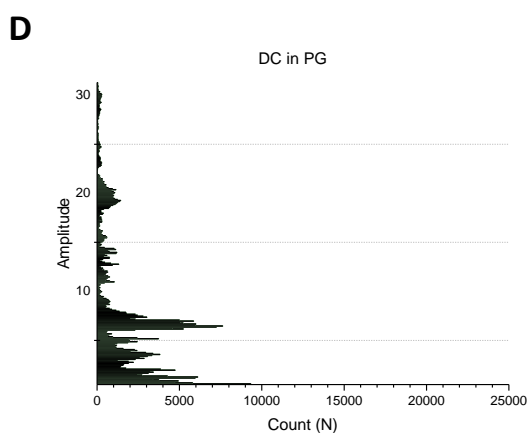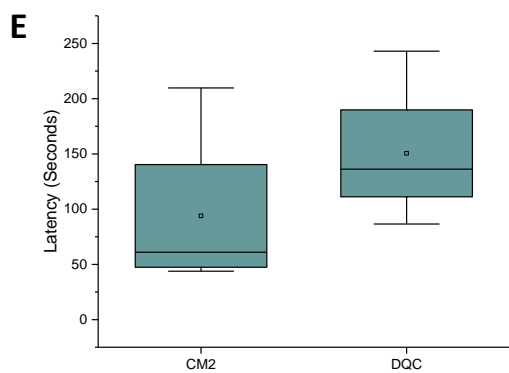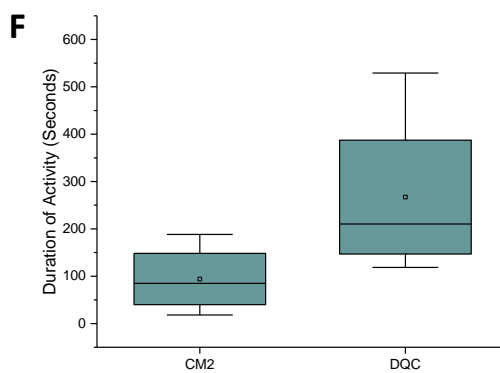

Supplement: FIG S2 [file msphere.00508-22-s0005.pdf]

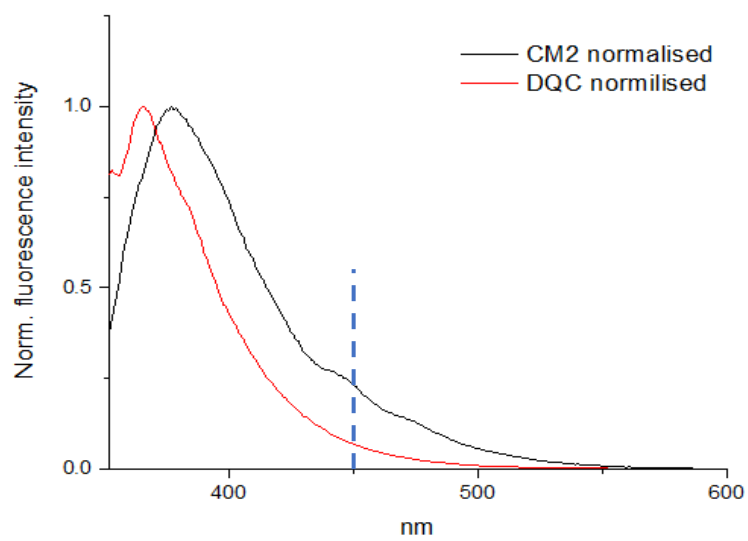

Supplement: FIG S3 [file msphere.00508-22-s0006.pdf]
