# Supplementary material for: Bolaamphiphile Analogues of 12-bis-THA Cl2 Are Potent Antimicrobial Therapeutics with Distinct Mechanisms of Action against Bacterial, Mycobacterial, and Fungal Pathogens
Source: mSphere. 2022 Dec 13;8(1):e00508-22. doi: 10.1128/msphere.00508-22 (PMC9942557; doi:10.1128/msphere.00508-22)
Supplement: TABLE S1 [file msphere.00508-22-s0001.pdf]

Antibiotic	MIC (µg/ml)					
	P. aeruginosa PAO1			*P. aeruginosa* NCTC 13437		
	No bola	Octa-bola	Octa-C10	No bola	Octa-bola	Octa-C10
Doxycyline	32	**128**	64	>256	>256	>256
Colistin	0.125-0.25	0.06	0.06	0.25-0.5	0.125	0.125-0.25
Ciprofloxacin	0.125	**1**	**1**	32	**>64**	**>64**
Meropenem	>2	>2	n.d.	>32	>32	>32
Ampicillin	>512	>512	n.d.	>512	>512	>512
Ceftazidime	2	2	n.d.	>128	>128	>128
Rifampin	16	16	8-16	16	16	8
