# Supplementary material for: Bolaamphiphile Analogues of 12-bis-THA Cl2 Are Potent Antimicrobial Therapeutics with Distinct Mechanisms of Action against Bacterial, Mycobacterial, and Fungal Pathogens
Source: mSphere. 2022 Dec 13;8(1):e00508-22. doi: 10.1128/msphere.00508-22 (PMC9942557; doi:10.1128/msphere.00508-22)
Supplement: TABLE S2 [file msphere.00508-22-s0002.pdf]

Combination	Strain	MIC alone (µg/ml)		MIC in combination (µg/ml)		FIC
		Bolalipid	Antibiotic	Bolalipid	Antibiotic	
Octa-C10 / streptomycin	*Ac. pleuropneumoniae* Shope	1	32 - 64	0.5	8	0.625 – 0.75
	Ac. pleuropneumoniae 2331	4 - 8	32	1	16	0.625 – 0.75
	Ac. pleuropneumoniae 2356	4 - 8	64	1 - 2	16 - 32	0.375 – 1
	Ac. pleuropneumoniae 3370	4 - 8	16 - 64	2	32	0.75 – 2.5
Octa-C10 / enrofloxacin	*Ac. pleuropneumoniae* Shope	1	0.125	0.25	0.0313	0.5
	Ac. pleuropneumoniae 2331	4 - 8	0.125	0.5	0.0625	0.563 – 0.625
	Ac. pleuropneumoniae 2356	4 - 8	0.125	0.5	0.0625	0.563 – 0.625
	Ac. pleuropneumoniae 3370	4 - 8	0.125	1	0.125	1.125 – 1.25
