# Supplementary material for: Bolaamphiphile Analogues of 12-bis-THA Cl2 Are Potent Antimicrobial Therapeutics with Distinct Mechanisms of Action against Bacterial, Mycobacterial, and Fungal Pathogens
Source: mSphere. 2022 Dec 13;8(1):e00508-22. doi: 10.1128/msphere.00508-22 (PMC9942557; doi:10.1128/msphere.00508-22)
Supplement: TABLE S3 [file msphere.00508-22-s0003.pdf]

Record	Mean count rate (kcps)	Derived count rate (kcps)	Z-Mean (nm)	PDI	Intercept	Pk 1 mean size (nm)	Pk 2 mean size (nm)	Pk 3 mean size (nm)
DQC	161 ± 19.8	1450 ± 179	246 ± 23.4	0.21 ± 0.02	0.96 ± 0.00	255 ± 15.4	3600 ± 3120	-
CM2	483 ± 76.5	483 ± 76.5	402 ± 57.4	0.44 ± 0.14	0.91 ± 0.01	248 ± 101	1930 ± 2990	-
Quino-bola	88.4 ± 14.3	88.4 ± 14.3	289 ± 37.1	0.43 ± 0.09	0.95 ± 0.02	140 ± 146	1940 ± 2820	3580 ± 3100
Penta-bola	302 ± 47.2	302 ± 47.2	283 ± 26.5	0.32 ±0.03	0.93 ± 0.00	266 ± 13.9	3580 ± 3100	
Hepta-bola	156 ± 25.1	156 ± 25.1	440 ± 12.6	0.26 ± 0.02	0.94 ± 0.02	426 ± 14.5	5300 ± 164	-
Octa-bola	371 ± 14.3	1320 ± 50.8	242 ± 6.5	0.19 ± 0.06	0.94 ± 0.01	261 ± 19.8	3540 ± 3070	-
Nanosphere	342 ± 140	1500 ± 75.6	62.6 ± 0.68	0.02 ± 0.01	0.95 ± 0.02	65.4 ± 0.83	-	-
